# Design characteristics of studies on medical practice variation of caesarean section rates: a scoping review

**DOI:** 10.1186/s12884-020-03169-3

**Published:** 2020-08-20

**Authors:** Maarten D H Vink, Piet J G M de Bekker, Xander Koolman, Maurits W van Tulder, Ralph de Vries, Ben Willem J Mol, Eric J E van der Hijden

**Affiliations:** 1 Department Health Sciences, Faculty of Science & Talma Institute, Vrije Universiteit, De Boelelaan 1085, 1081 HV Amsterdam, the Netherlands; 2 Department of Obstetrics and Gynaecology, University Medical Center Groningen, Groningen, the Netherlands; 3 Medical Library, Vrije Universiteit Amsterdam, Amsterdam, The Netherlands; 4 Department of Obstetrics and Gynaecology, Monash University, Clayton, Victoria Australia; 5 Zilveren Kruis Health Insurance, Leusden, The Netherlands

**Keywords:** Caesarean section, Medical practice variation, Study design characteristics

## Abstract

**Background:**

Medical practice variation in caesarean section rates is the most studied type of practice variation in the field of obstetrics and gynaecology. This has not resulted in increased homogeneity of treatment between geographic areas or healthcare providers. Our study aim was to evaluate whether current study designs on medical practice variation of caesarean section rates were optimized to identify the unwarranted share of practice variation and could contribute to the reduction of unwarranted practice variation by meeting criteria for audit and feedback.

**Methods:**

We searched PubMed, Embase, EBSCO/CINAHL and Wiley/Cochrane Library from inception to March 24th, 2020. Studies that compared the rate of caesarean sections between individuals, institutions or geographic areas were included. Study design was assessed on: selection procedure of study population, data source, case-mix correction, patient preference, aggregation level of analysis, maternal and neonatal outcome, and determinants (professional and organizational characteristics).

**Results:**

A total of 284 studies were included. Most studies (64%) measured the caesarean section rate in the entire study population instead of using a sample (30%). (National) databases were most often used as information source (57%). Case-mix correction was performed in 87 studies (31%). The Robson classification was used in 20% of the studies following its endorsement by the WHO in 2015. The most common levels of aggregation were hospital level (35%) and grouped hospitals (35%) e.g. private versus public. The percentage of studies that assessed the relationship between variation in caesarean section rates and maternal outcome was 9%, neonatal outcome 19%, determinants (professional and organizational characteristics) 21% and patient preference 2%.

**Conclusions:**

Study designs of practice variation in caesarean sections varied considerably, raising questions about their appropriateness. Studies focused on measuring practice variation, rather than contributing to the reduction of unwarranted practice variation. Future studies should correct for differences in patient characteristics (case-mix) and patient preference to identify unwarranted practice variation. Practice variation studies could be used for audit and feedback if results are presented at lower levels of aggregation, and appeal to intrinsic motivation of physicians, for example by including the health effects on mother and child.

## Background

The caesarean section has been the most performed surgical procedure worldwide for many decades [1]. It has been extensively studied, both to optimize treatment [2] and to understand why deviations from optimal treatment occur [3]. This long-term popularity has not resulted in evidence based homogeneous treatment between geographic areas or healthcare providers [4, 5]. The magnitude of the variation has raised questions about regional differences in quality of healthcare, especially in countries with similar resources [6]. Moreover, 40 years of study on practice variation shows no trend of increasing regional – let alone worldwide – conformity [4].

Variation in medical practice can be divided in warranted and unwarranted practice variation [7]. Variation is warranted when it results from variation in need, for example due to varying rates of extreme premature deliveries. Extremely preterm deliveries are centralized at institutions with the highest expertise of neonatal care, as it yields the most optimal outcome [8]. These institutions may deviate from the national average, as they serve a high-risk population.

Medical practice variation is unwarranted if it cannot be explained by patient characteristics or patient preference [9, 10]. To identify unwarranted practice variation, studies should compare study groups that are comparable in terms of relevant patient characteristics or make them comparable through careful case-mix correction [11]. Patient preference is important when both modes of delivery – vaginal delivery and caesarean section – are an acceptable option. Variation of caesarean section rates is desirable to allow for differences in patient preference across healthcare providers and random or unmeasured differences in need of having a caesarean section. When a reported rate deviates more from an acceptable range, differences may less likely be attributable to differences in patient preference, as both over- and undertreatment of caesarean sections harms mother and child [12].

It is therefore likely that quality of healthcare for mother and child can be improved by reducing unwarranted practice variation of caesarean sections. Sufficient evidence on risks and benefits of caesarean sections may help to reduce variation. Higher quality evidence will result in better guidance on the optimal caesarean section rate for specific obstetric conditions [13]. Subsequently, up-to-date clinical guidelines and clinical support systems may facilitate clinicians to implement recent evidence [14]. Finally, shared decision making should be incorporated in daily clinical practice to empower patients to decide what suits them best [15].

Improving the process of generating evidence, implementing clinical guidelines and incorporating shared decision making requires healthcare professionals to change their clinical behavior, which is complex. Audit and feedback is a non-clinical intervention that supports change of clinical behavior [16]. Literature shows that healthcare professionals may be encouraged with information relevant to them. One example is performance feedback on a low level of aggregation, i.e. group or individual level [17]. Another is to tap into the intrinsic motivation to do well for patients. Evidence shows that unnecessary caesarean sections cause an increase in maternal death rates and may affect infant health negatively [12]. Monitoring and reporting mother and child health may motivate change to reduce unnecessary caesarean sections [18]. Audit and feedback has been put forward as a way through which research can contribute to the reduction of practice variation [19], but it is unclear whether current research designs of studies on medical practice variation of caesarean section rates can be used for this purpose.

Medical practice variation in caesarean section rates has been extensively studied [20]. Studies started as early as 1979 [21] and the interest for the topic has remained strong [22]. However, (unwarranted) medical practice variation of caesarean section rates has not been reduced [4]. The objective of this scoping review is to evaluate whether studies on medical practice variation of caesarean section rates have used an optimal study design to identify unwarranted practice variation and - when identified – can also be used for audit and feedback to contribute to the reduction of unwarranted practice variation.

## Methods

To evaluate the characteristics of all caesarean section variation studies, we opted for a scoping review. We followed the PRISMA statement [23]. The research protocol was not published.

### Search strategy

We searched studies that compared caesarean section rates between (individual) healthcare professionals, hospitals, groups of hospitals, or geographic areas. A comprehensive search was performed in collaboration with a medical librarian in the bibliographic databases PubMed, Embase, EBSCO/CINAHL and Wiley/Cochrane Library from inception up to March 24th, 2020. The following terms were used (including synonyms and closely related words) as index terms or free-text words: “Practice Patterns”, “Caesarean Section”. The free-text term “Caesarean Section” was only used in titles. The MeSH-term “Practice Patterns” includes several descriptions of practice variation. The search strategy was performed without date or language restriction. The full search strategies for all databases can be found in additional file 1.

### Study selection

All studies that reported on any variation in caesarean section rates between (individual) healthcare professionals, hospitals, groups of hospitals, or geographic areas were included. We included any type of study design; i.e., cross-sectional study designs, and both prospective and retrospective longitudinal studies.

### Exclusion criteria

We excluded studies that were not published in English and studies that did not publish data on variation of caesarean section rates between healthcare professionals, hospitals, groups of hospitals, or geographic areas.

### Process of study identification and selection

Titles and abstracts were downloaded and entered in EndNote (version X8.1). Duplicates were removed. Two researchers (MV and PdB) screened titles and abstracts. The researchers independently decided whether to include the article for full text screening. Disagreement was resolved by consensus. If no consensus could be achieved a third researcher made the decision (EvdH). Full text screening was performed by two researchers who decided independently whether to include the article or not (MV and PdB). In case of disagreement the third researcher (EvdH) decided whether the article met the pre-defined inclusion criteria. Data on the design characteristics of the studies were extracted by one researcher (MV). These data were randomly cross-checked by a second researcher (PdB).

### Data extraction

To describe the variation of caesarean section rates, we scored the minimum and maximum caesarean section rate. When caesarean section rates of multiple years were reported the rate of the most recent year was used. As an indicator for the risk of selection bias we scored the selection of study population. We differentiated between the use of a study sample or the entire study population, to estimate the caesarean section rate. The use of a study sample was considered as a high risk of selection bias if the study lacked a description of the sampling frame. The assessment of the caesarean section rate of the entire study population was considered as a low risk of selection bias. To indicate the risk of information bias, we differentiated between the use of electronic patient files (EPF), a (national) database or questionnaires. The use of EPF and databases were considered as low risk of bias, as the information was collected by attending healthcare professionals.

### Identification of unwarranted practice variation

To identify whether studies distinguished between warranted and unwarranted practice variation we scored whether case-mix correction was performed and if patient preference was taken into account.

No restriction was imposed on the method of case-mix correction. Examples of case-mix corrections include calculating an adjusted or expected caesarean section rate, reporting stratified odds ratios by patient characteristics or using logistic regression to adjust for patient characteristics. We extracted which variables were used for case-mix correction and whether the Robson classification was used. The latter is the system proposed by the World Health Organisation (WHO) and the International Federation of Obstetrics and Gynaecology (FIGO) to classify caesarean section case-mix [24, 25].

No restriction was imposed on how patient preference was measured. Measuring patient preference requires additional data collection. This could be unfeasible for large cohort studies unless a truly random sample of sufficient size is used. We assessed whether all studies took patient preference into account, registered the cohort size and noted how patient preference was measured. If patient preference was measured, we assessed whether a sample was used and whether it was random.

### Usefulness for audit and feedback

To evaluate whether the studies were able to provide healthcare professionals’ feedback on their clinical behavior in order to reduce unwarranted practice variation, we extracted the aggregation level of analysis that was used and differentiated between: individual physician, group of physicians, hospital, hospital category, region or country. Similarly, we scored whether maternal and neonatal outcomes were measured, as outcome reporting informs healthcare professionals on their clinical performance. We extracted all reported variables.

In addition, we extracted several explanatory factors that might contribute to unwarranted practice variation, including organizational characteristics (i.e. profit-status or teaching-status of the hospital) and physician characteristics (i.e. physician gender and age). Furthermore, we scored whether studies analysed financial consequences of (unwarranted) practice variation of caesarean section rates.

## Results

**Fig. 1:**
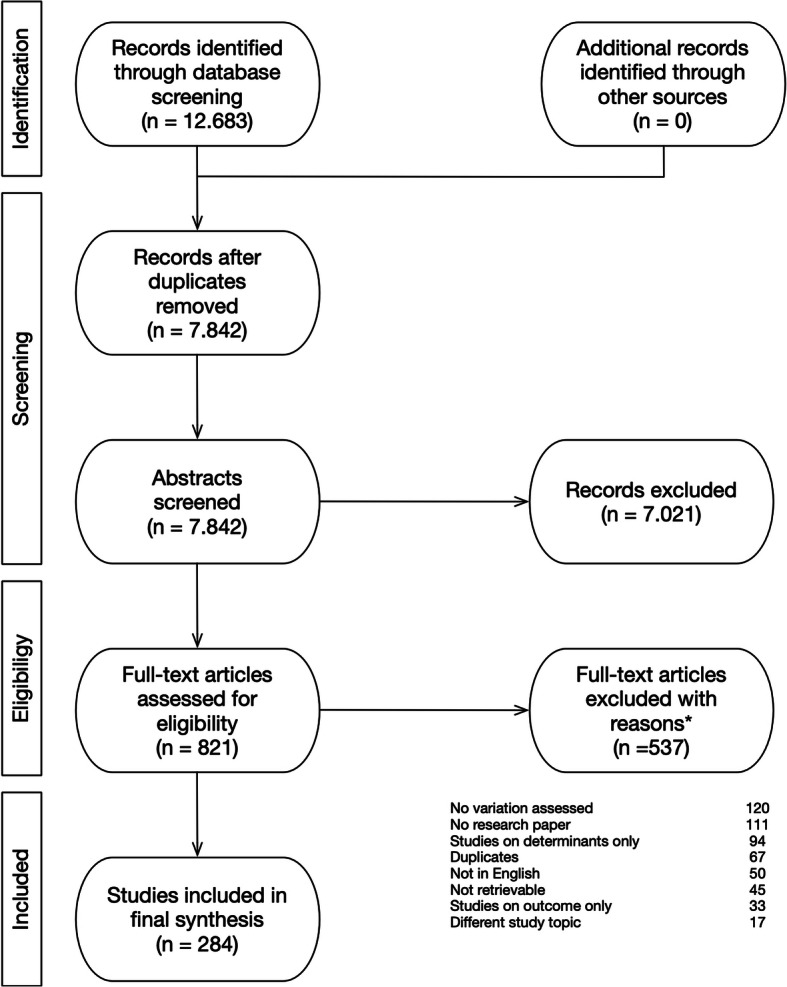
PRISMA flowchart

The process of study identification and selection is schematically presented according to the PRISMA statement in Fig. 1. A total of 12.683 records were identified: 3.967 from PubMed, 5.790 from Embase, 1.153 from Cochrane and 1.773 from Cinahl. After duplication we screened 7.842 abstracts for eligibility. In total 821 articles were selected for full-text screening. We excluded 537 studies. The reasons for exclusion are presented in Fig. 1. In total 284 studies met the inclusion criteria and were included. The included studies and their design characteristics and reported variation in caesarean section rates are listed in additional file 2.

The included studies were published between 1979 and 2020. The cohorts that were studied varied between 94 and more than 100 million women. Most studies analyzed variation of caesarean section rates in the United States (75 studies) followed by Brazil (22 studies) and Australia (17 studies). The number of studies per country are shown in additional file 3. A wide variation in caesarean section rates is reported. Some Sub-Saharan regions perform caesarean sections in less than 1% of the deliveries. By contrast, the reported caesarean section rate of some municipalities in Brazil reached 97%.

**Fig. 2:**
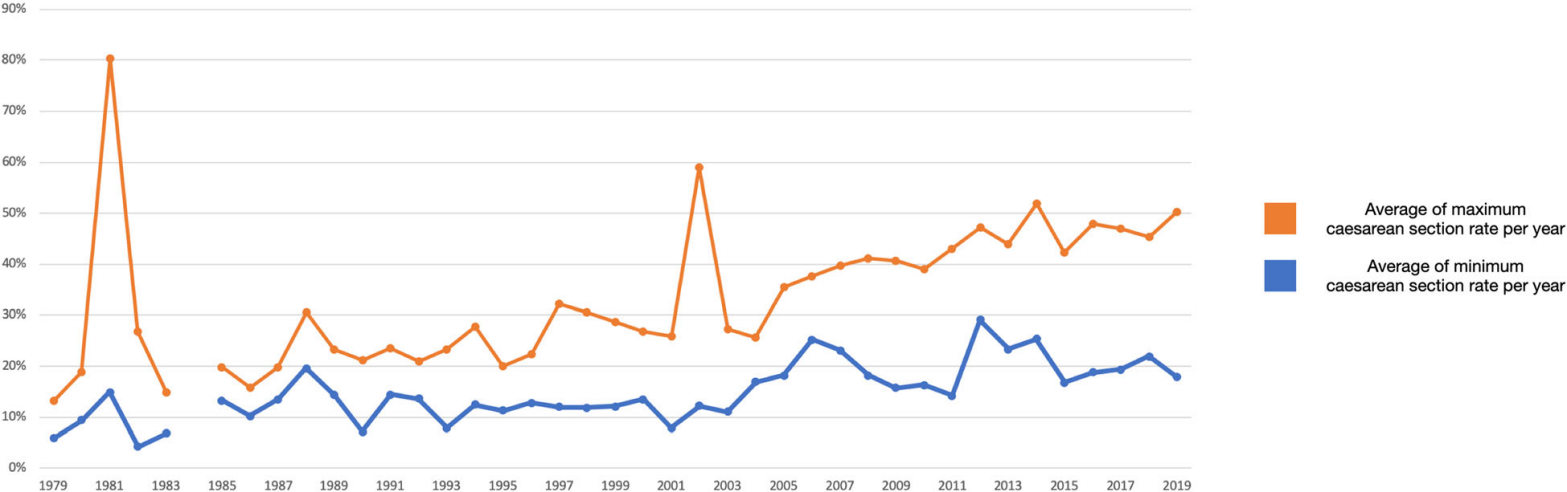
Average of the reported minimum and maximum caesarean section rate per year. * 2020 is not presented in Fig. 2, as only studies are only included until 24th of March 2020

The variation of caesarean section rates did not decrease over time. Figure 2 shows the average reported minimum and maximum caesarean section rates per year. The outlier in 1981 is one study that reported variation between four hospitals in Rio de Janeiro. In one private hospital more than 80% of the women delivered by a caesarean section. In 2002 a similar situation occurred: only two studies were included, both reporting on variation in India.

In 85 studies (30%) a sample of the study population was used to estimate the caesarean section rate. The majority of the studies (182 studies, 64%) measured the mode of delivery of the entire study population. Both methods were used in eight studies (3%) and the selection frame was unclear in nine studies (3%). An example of a study in which both methods were used was the analysis of variation in caesarean section rates between countries. Some country estimates were based on a sample of their population, while others were based on registries of the entire population. In the sample-based studies, 41 studies (48%) defined a selection frame designed to select a representative sample.

The majority of studies used data from registries such as (national) databases (161 studies, 57%) or electronic patient files (27 studies, 10%). Questionnaires were used in 51 studies (18%), e.g. the Demographic and Health Survey (DHS) that was used in 25 (9%) studies. Such questionnaires were sent to a sample of households in order to collect information on live births of the past years. In 45 studies (16%) multiple data sources were used, or the data source was not described.

### Identification of unwarranted practice variation

Case-mix correction was performed in 87 studies (31%). The variables that were used for case-mix correction are shown in additional file 4. Baseline patient characteristics were observed in 80 studies (28%). Some studies did not describe patient characteristics per cohort but did perform a correction for maternal or neonatal characteristics. Many different maternal (86) and obstetric (128) variables were used as baseline characteristic or for case-mix correction. Age (118), parity (74), gestational age (66), birthweight (61), and maternal education level (48) are the characteristics that were used most often. More than half of the variables were only used in one or two studies.

**Fig. 3:**
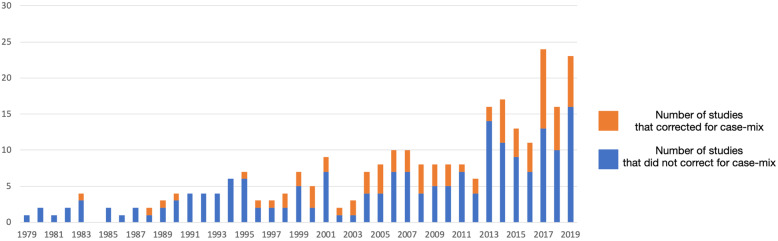
Number of studies per year with and without case-mix correction. * 2020 is not presented in Fig. 3, as only studies are only included until 24th of March 2020

To reduce this heterogeneity and to increase the quality of case-mix correction, the WHO in 2015 recommended to use the Robson Classification as the standard for case-mix correction for studies on caesarean section rates [24]. In the period 2015–2020 19 out of 93 studies used the Robson Classification (20%). Four of these studies (21%) did perform additional case-mix correction by using specific patient characteristics. The WHO notes that the Robson classification should especially be used when comparing caesarean section rates between healthcare facilities, or within healthcare facilities over time [24]. In the period 2015–2020 67 studies compared caesarean section rates between healthcare providers (individual level, group of physicians, hospital level and hospital category) of which 16 used the Robson Classification (24%). Within the same period 4 of the 41 studies that compared caesarean section rates between geographic areas used the Robson Classification (10%). The percentage of studies that corrected for case-mix did not change over time. Figure 3 shows the number of studies that corrected for case-mix by year.

**Fig. 4:**
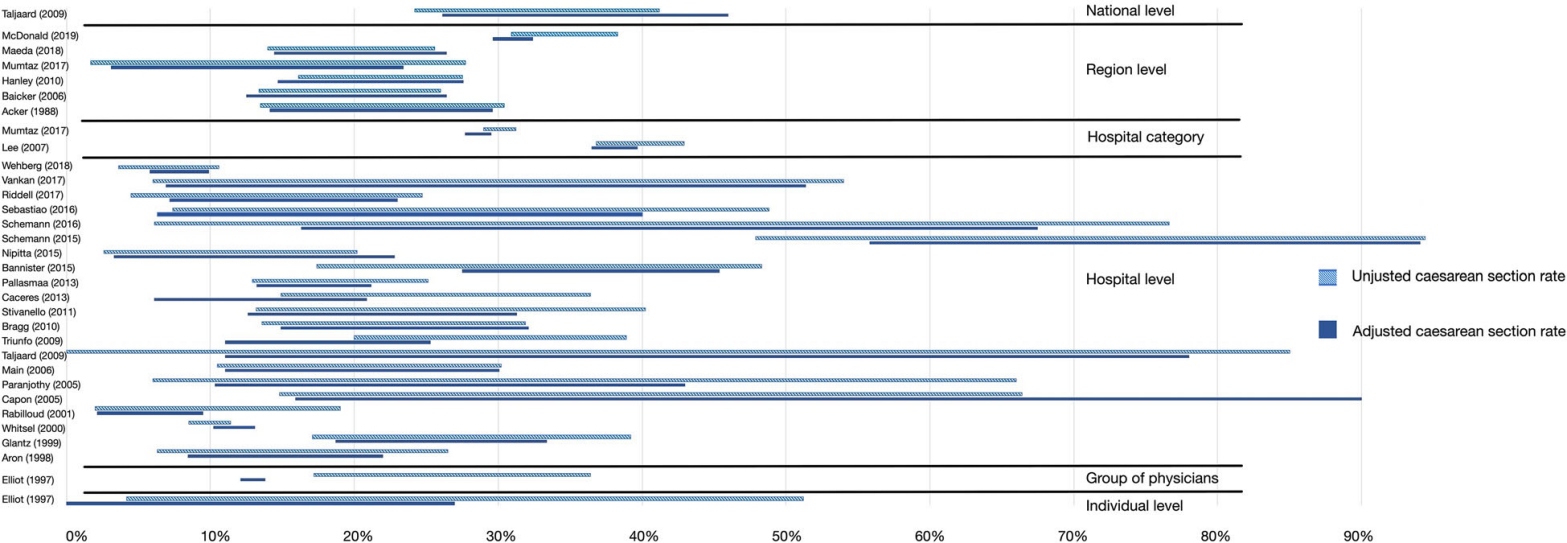
The effect of case-mix correction on different aggregation levels

The effect of case-mix correction is shown in Fig. 4. Of the 87 studies (31%) that corrected for case-mix, 29 studies (33%) calculated an adjusted caesarean section rate. Figure 4 shows these rates per study categorized per aggregation level. The remaining 58 studies (67%) calculated an expected caesarean section rate, reported stratified odds ratios by patient characteristics or used logistic regression to adjust for patient characteristics. At provider level (individual physician, group of physicians and hospital level) 35% of the studies and at geographic level (regional or national) 23% of the studies corrected for case-mix. Figure 4 shows that at the provider level case-mix correction had a substantial impact on the provider caesarean section rate. At the geographic level the impact of case-mix correction was comparatively small.

Six studies (2%) took patient preference into account. These studies did not assess variation of caesarean section rates for a specific obstetric condition (for instance patients with a history of caesarean section) in which a caesarean section and vaginal delivery are both an acceptable option [15]. All studies (6) measured patient preference by questionnaires that were handed to mothers that gave birth between one day and 2 years prior to the survey. Patient preference was assessed by posing a variety of questions on the reason of the caesarean section and perceived influence on decision making. No studies reported the quality of decision making. In 4 of the 6 studies (67%) a sample of the study population was used to measure patient preference; samples varied between 512 and 8.717 women and three studies used a predefined sampling frame.

### Usefulness for audit and feedback

Table 1 shows the study characteristics by aggregation level. The majority of the studies (75%) used one aggregation level. A minority used two (24%) and only three studies (1%) used three aggregation levels. Healthcare providers (individual physicians, group of physicians, hospitals or hospital categories) were compared in 200 studies (70%) and geographic areas (national or regional) in 124 studies (44%). Hospitals (35%) and grouped hospitals (35%) e.g. private vs. public hospitals were most often used as aggregation level of analysis. Medical practice variation of caesarean section rates was least often studied at the level of the individual physician (4%) and group of physicians (4%). No clear time trend was observed in the choice of aggregation level or trend in observed variation based on the level of aggregation. The largest variation in caesarean section rates is reported on both the lowest – i.e. individual – level and the highest – i.e. international – level of aggregation.

**Table 1:** Study characteristics by aggregation level

	Individual level	Group of physicians	Hospital level	Hospital category	Region	National
Number of studies	12	11	99	98	95	30
Average cohort size	5.433	26.483	154.370	385.274	1.032.698	680.265
Median cohort size	5.559	2.841	23.236	74.074	259.627	133.293
Entire population measured (instead of sample)	100%	91%	79%	59%	64%	30%
Reported variation in caesarean section rate (average of studies)	8–36%	15–26%	13–37%	25–44%	15–34%	12–37%
Reported variation in caesarean section rate (median of studies)	6–35%	11–29%	11–33%	23–37%	15–30%	11–40%
Case-mix correction	4 (33%)	3 (27%)	42 (42%)	28 (29%)	22 (23%)	6 (20%)
Medical outcome	9 (75%)	5 (45%)	24 (24%)	11 (11%)	12 (13%)	7 (23%)

Neonatal outcomes were captured in 53 (19%) and maternal outcomes in 25 (9%) of the studies. All variables that were used to measure these outcomes are listed in additional file 4. Many different variables were used to measure neonatal (26) and maternal (13) outcomes. Neonatal mortality (27), Apgar score (25), maternal mortality (13), NICU admission (12) and haemorrhage (6) are outcomes that were measured most often. Half of the outcome variables were used in just one single study. Table 1 shows the numbers of studies per aggregation level that took neonatal and maternal outcome into account. Neonatal and maternal outcomes were most often reported if variation of caesarean sections was studied at the level of the individual physicians.

### Determinants and financial consequences

A limited number of studies explored determinants to explain medical practice variation of caesarean section rates in an additional analysis. Hospital characteristics or physician characteristics were used in 60 studies (21%) to explain differences in caesarean section rates. The variables that were used are listed in additional file 4. Financial consequences of (unwarranted) variation of caesarean section rates were calculated in six (2%) studies.

## Discussion

Almost four decades have past and 283 studies were published following Opit’s first report on variation of caesarean section rates between geographic areas in Australia. Clearly the issue raised by the first studies has not lost its sense of urgency among researchers, nor for the funders of research, because the magnitude of unwarranted variation was considered problematic [26] and remained stable over time [27]. While previous reviews narrowed their focus on measuring variation between geographic areas [28, 29] or studied the difference between public and private hospitals [30], the focus of this review was on the presence of study characteristics that may help to reduce unwarranted variation of caesarean section rates.

### Strengths and limitations

A strength of our review was the systematic search and selection procedure, which allowed us to identify (almost) all studies on medical practice variation that compared caesarean section rates. This resulted in a large number of included studies. A second strength is the high level of detail of our analysis. The selection of the individual variables that were used for case-mix correction, outcomes or determinants enabled us to present an in-depth overview of study characteristics.

Several limitations should be addressed. First, we aimed to describe study characteristics of all relevant published studies - i.e. irrespective of the quality - in the English language and therefore did not perform a quality assessment of the included studies. Second, we were unable to retrieve all publications that were selected for full-text screening. In order to limit the number of missing articles we contacted the authors of missing articles by email or through ResearchGate. However, this was not successful for 45 of the 821 studies.

### Interpretation

First, we appraised whether studies were designed to distinguish between warranted and unwarranted practice variation by performing case-mix correction and analysing patient preference. Case-mix correction is an essential aspect of the quality of studies on practice variation, because without case-mix correction it remains unclear what share of the variation is attributable to health differences between populations, and thus to what extent the variation is warranted. Our results show that only 35% of the studies that compared caesarean section rates between healthcare providers performed case-mix correction. Case-mix correction was performed by calculating adjusted rates, expected rates, stratified odds ratios or logistic regression analysis. Over time we observed no improvement in the performance of case-mix correction.

Patient preference, another important aspect to identify unwarranted practice variation was only measured in six studies (2%). Without the assessment of patient preference, it remains unclear whether practice variation can be explained by differences in the outcome of shared decision making. This is especially important when both modes of delivery – vaginal delivery and caesarean section – are an acceptable option. Patients with a history of caesarean section, breech or twin delivery are examples in which information on patient preference is necessary to differentiate between warranted and unwarranted medical practice variation [31–33]. Our results show that none of studies that assessed variation in these specific obstetric situations patient preference was analysed. To improve the identification of unwarranted practice variation future studies should not only measure patient preference but should focus on the implementation of shared decision making. Recent literature shows that several shared decision making measures are available which could be included in the study design of medical practice variation studies [34].

To improve comparability both within healthcare facilities and between them, Robson proposed what later came to be known as the Robson Classification System for assessing, monitoring and comparing caesarean section rates in 2001. The WHO and the FIGO jointly endorsed this classification as the international standard for case-mix correction [24, 25]. Our data show that following the publication of this guideline by the WHO in 2015, the Robson classification was only used in 16 (24%) of all studies comparing providers and in 4 studies (10%) comparing regions. Literature shows that case-mix correction can be improved even more if additional patient characteristics are considered [35]. Only 21% of the studies that were published between 2015 and 2020 and used the Robson classification did perform additional case-mix correction (i.e. age adjusted caesarean section rates within Robson groups). Studies that performed case-mix correction (with or without Robson classification) used a wide variation of maternal and obstetric characteristics. To identify unwarranted practice variation, we advise to at least use the Robson Classification and to standardise variables for additional case-mix correction. A Delphi procedure can help obstetricians and midwives to reach consensus on which variables to use [36].

The necessity to which case-mix correction is needed depends on the level of aggregation. The lower the level of aggregation the more case mix correction contributes to a valid description of clinical performance [37]. Health care providers often operate in a network and treat a subset of the entire population. That subset is more likely to differ between providers as they get more specialized and the case-mix differences may justify differences in caesarean section rates [38]. This requires that studies aimed at lower levels of aggregation place more emphasis on case-mix correction, reporting standards and appropriate small number statistics [39].

Once case-mix has been appropriately controlled for, future studies should aim to decompose regional unwarranted variation into lower levels of aggregation. This decomposition allows regional variation to be attributed to health care providers. However, disaggregation of contributions to lower levels of aggregation is not without major risk on its own. As groups of physicians get smaller, their client-base may diverge more from the regional average, for example due to specialization, reporting differences or chance. Within providers, e.g. hospitals, further disaggregation to the physician level could help identify individual contributions. While the lower number of observations may harm both validity and reliability, they may also provide a stronger stimulus for change if the information is interpreted intelligently and presented in a motivating environment.

Stimulus for change might be further enhanced when outcome reporting becomes integrated in future study designs. Healthcare professionals are intrinsically motivated to deliver the best healthcare for their patients [40]. Reporting outcomes in case-mix corrected feedback information directly stimulates the intrinsic motivation to improve outcome for patients. If it becomes visible that increased caesarean section rates do not yield improved maternal and neonatal outcomes, professionals might be encouraged to adapt clinical behavior. Our results show that the wide variation in outcome variables demands consensus and standardization. Studies will become more comparable and better interpretable when a standard set of outcomes is used. For maternal infectious morbidity outcomes after caesarean delivery a core outcome set (COS) is developed [41]. We encourage to develop a COS for neonatal outcomes after caesarean section.

## Conclusion

Forty years of research on caesarean section rates have been unable to reduce unwarranted practice variation. Our study shows that most studies do not meet the criteria to identify unwarranted practice variation and cannot be used for audit and feedback. To contribute to the reduction of unwarranted practice variation future studies should correct for differences in patient characteristics and patient preference, present results at low levels of aggregation, and appeal to intrinsic motivation by including the health effects on mother and child.

## Supplementary information

**Additional file 1.** Search strategies. Additional file 1 contains the search strategies that were used for the databases PubMed, Embase, EBSCO/CINAHL and Wiley/Cochrane Library.

**Additional file 2.** List of included studies. Additional file 2 contains a list of all studies that were included in this scoping review. Per study the results are summarized. We used the following definitions for the independent variables used in the table: - Year: year of publication. - Author: first author of study included. - Title: title of study included. - Study period: years from which caesarean section rates were reported. In example, if a researcher performed a (questionnaire) survey in 2000 and included deliveries from 2 y prior to the survey we reported study period 1998–2000. - Caesarean section rate: unadjusted caesarean section rate of most recent year reported. - Cohort size: the cohort size from which the caesarean section rate is calculated. If caesareans section rates from multiple years were reported, we noted specifically the cohort size of the cohort that was used to calculate the most recent caesarean section rate. - Data source: data source that was used by the authors to calculate the reported caesarean section rate. It is reported as “other” if data source is unknown or multiple data sources are used. - Case-mix correction: the study reported an adjusted caesarean section rate, expected caesarean section rate, reported stratified odds ratios by patient characteristics or used logistic regression to adjust for patient characteristics (Y/N). - Aggregation level: aggregation level of analysis. - Outcome: outcomes (maternal or neonatal) were noted (Y/N). - Determinants: organisational or physician characteristics were used to explain reported difference in caesarean section rates between healthcare professionals, hospitals, groups of hospitals or geographic areas (Y/N).

**Additional file 3.** Studies per country. Additional file 3 describes the number of studies on medical practice variation of caesarean section rates that were conducted in each country.

**Additional file 4.** Variables. Additional file 4 summarizes all the variables that were used in the included studies: - Patient characteristics: variables that were used as baseline characteristic or for case-mix correction. - Outcome: variables on maternal and neonatal outcome. - Organization or physician characteristics: characteristics that were used as determinant to explain differences in caesarean section rates.

## Data Availability

The dataset used and/or analysed during the current study are available from the corresponding author on reasonable request.

## Abbreviations

COS: Core Outcome Set
DHS: Demographic and Health Survey
EPF: Electronic Patient Files
FIGO: International Federation of Gynecology and Obstetrics
WHO: World Health Organisation
