# Supplementary material for: Design characteristics of studies on medical practice variation of caesarean section rates: a scoping review
Source: BMC Pregnancy Childbirth. 2020 Aug 20;20:478. doi: 10.1186/s12884-020-03169-3 (PMC7441547; doi:10.1186/s12884-020-03169-3)
Supplement: Supplementary file 1 — Additional file 1. Search strategies. Additional file 1 contains the search strategies that were used for the databases PubMed, Embase, EBSCO/CINAHL and Wiley/Cochrane Library. [file 12884_2020_3169_MOESM1_ESM.docx]

# Additional file 1: Search strategies

A literature search was performed based on the Preferred Reporting Items for Systematic Reviews and Meta-Analyses (PRISMA)-statement ([www.prisma-statement.org](http://www.prisma-statement.org)).

To identify all relevant publications we conducted systematic searches in the bibliographic databases PubMed, Embase.com, Cochrane Library (Wiley) and CINAHL (Ebsco) from inception up to March 24, 2020, in collaboration with a medical information specialist. The following terms were used (including synonyms and closely related words) as index terms or free-text words: “Practice Patterns”, “Cesarean Section”. Free-text “Cesarean Section” in title only.

The references of the identified articles were searched for relevant publications. Duplicate articles were excluded. All languages were accepted. The full search strategies for all databases is described below.

**PubMed Session Results (24 March 2020)**

| Search | Query | Items found |
| --- | --- | --- |
| #3 | **#1 AND #2** | 3,967 |
| #2 | "Cesarean Section/statistics and numerical data"[Majr] OR "Cesarean Section/trends"[Majr] OR cesarea*[ti] OR caesarea*[ti] OR "c section"[ti] OR "c sections"[ti] OR (abdominal[ti] AND deliver*[ti]) | 24,737 |
| #1 | ("Practice Patterns, Physicians'"[Mesh] OR practice pattern*[tiab] OR variation*[tiab] OR variance*[tiab] OR variabil*[tiab] OR rate*[tiab] OR trend*[tiab]) AND (hospital*[tw] OR interhospital[tiab] OR institut*[tiab] OR interinstitutional[tiab] OR obstetr*[tiab] OR interobstetr*[tiab] OR laborist*[tiab] OR interlabor*[tiab] OR inter-labor*[tiab] OR geographic*[tiab] OR regional[tiab] OR state[tiab]) | 776,539 |

**Embase.com Session Results (24 March 2020)**

| Search | Query | Items found |
| --- | --- | --- |
| #3 | **#1 AND #2** | 5,790 |
| #2 | cesarea*:ti OR caesarea*:ti OR 'c section':ti OR 'c sections':ti OR (abdominal:ti AND deliver*:ti) | 30,438 |
| #1 | ('clinical practice'/exp OR 'practice pattern*':ab,ti,kw OR variation*:ab,ti,kw OR variance*:ab,ti,kw OR variabil*:ab,ti,kw OR rate*:ab,ti,kw OR trend*:ab,ti,kw) AND (hospital*:ab,ti,kw,de OR interhospital:ab,ti,kw OR institut*:ab,ti,kw OR interinstitutional:ab,ti,kw OR obstetr*:ab,ti,kw OR interobstetr*:ab,ti,kw OR laborist*:ab,ti,kw OR interlabor*:ab,ti,kw OR 'inter-labor*':ab,ti,kw OR geographic*:ab,ti,kw OR regional:ab,ti,kw OR state:ab,ti,kw) | 1,264,885 |

**Wiley / Cochrane Library Session Results (24 March 2020)**

| Search | Query | Items found |
| --- | --- | --- |
| #3 | **#1 AND #2** | 1,153 |
| #2 | cesarea*:ti or caesarea*:ti or (c NEXT section):ti or (c NEXT sections):ti or (abdominal:ti and deliver*:ti) | 7,090 |
| #1 | (((practice NEXT pattern*):ab,ti,kw or variation*:ab,ti,kw or variance*:ab,ti,kw or variabil*:ab,ti,kw or rate*:ab,ti,kw or trend*:ab,ti,kw) and (hospital*:ab,ti,kw or interhospital:ab,ti,kw or institut*:ab,ti,kw or interinstitutional:ab,ti,kw or obstetr*:ab,ti,kw or interobstetr*:ab,ti,kw or laborist*:ab,ti,kw or interlabor*:ab,ti,kw or (inter NEXT labor*):ab,ti,kw or geographic*:ab,ti,kw or regional:ab,ti,kw or state:ab,ti,kw)) | 100,917 |

**CINAHL (Ebsco) Session Results (24 March 2020)**

| Search | Query | Items found |
| --- | --- | --- |
| S3 | **S1 AND S2** | 1,773 |
| S2 | TI (cesarea* OR caesarea* OR "c section" OR "c sections" OR (abdominal AND deliver*)) | 10,772 |
| S1 | (MH "Practice Patterns") OR TI (("practice pattern*" OR variation* OR variance* OR variabil* OR rate* OR trend*) AND (hospital* OR interhospital OR institut* OR interinstitutional OR obstetr* OR interobstetr* OR laborist* OR interlabor* OR "inter-labor*" OR geographic* OR regional OR state)) OR AB (("practice pattern*" OR variation* OR variance* OR variabil* OR rate* OR trend*) AND (hospital* OR interhospital OR institut* OR interinstitutional OR obstetr* OR interobstetr* OR laborist* OR interlabor* OR "inter-labor*" OR geographic* OR regional OR state)) OR SU (("practice pattern*" OR variation* OR variance* OR variabil* OR rate* OR trend*) AND (hospital* OR interhospital OR institut* OR interinstitutional OR obstetr* OR interobstetr* OR laborist* OR interlabor* OR "inter-labor*" OR geographic* OR regional OR state)) | 250,603 |
