# Supplementary material for: Design characteristics of studies on medical practice variation of caesarean section rates: a scoping review
Source: BMC Pregnancy Childbirth. 2020 Aug 20;20:478. doi: 10.1186/s12884-020-03169-3 (PMC7441547; doi:10.1186/s12884-020-03169-3)
Supplement: Supplementary file 2 — Additional file 2. List of included studies. Additional file 2 contains a list of all studies that were included in this scoping review. Per study the results are summarized. We used the following definitions for the independent variables used in the table: - Year: year of publication. - Author: first author of study included. - Title: title of study included. - Study period: years from which caesarean section rates were reported. In example, if a researcher performed a (questionnaire) survey in 2000 and included deliveries from 2 y prior to the survey we reported study period 1998–2000. - Caesarean section rate: unadjusted caesarean section rate of most recent year reported. - Cohort size: the cohort size from which the caesarean section rate is calculated. If caesareans section rates from multiple years were reported, we noted specifically the cohort size of the cohort that was used to calculate the most recent caesarean section rate. - Data source: data source that was used by the authors to calculate the reported caesarean section rate. It is reported as “other” if data source is unknown or multiple data sources are used. - Case-mix correction: the study reported an adjusted caesarean section rate, expected caesarean section rate, reported stratified odds ratios by patient characteristics or used logistic regression to adjust for patient characteristics (Y/N). - Aggregation level: aggregation level of analysis. - Outcome: outcomes (maternal or neonatal) were noted (Y/N). - Determinants: organisational or physician characteristics were used to explain reported difference in caesarean section rates between healthcare professionals, hospitals, groups of hospitals or geographic areas (Y/N). [file 12884_2020_3169_MOESM2_ESM.docx]

# Additional file 2: list of included studies

| **Year** | **Author** | **Title** | **Study period** | **Cesarean section rate** | **Cohort size** | **Sample used** | **Data source** | **Case-mix correction** | **Aggregation level** | **Outcome measured** | **Determinants** |
| --- | --- | --- | --- | --- | --- | --- | --- | --- | --- | --- | --- |
| 1979 | Opit | Caesarean-section rates in Australia: a population-based audit | 1963 – 1978 | 5.9% - 13.3% | 84.283 | Yes | Database | No | Region | Yes | Yes |
| 1980 | Placek | Trends in cesarean section rates for the United States. 1970--78 | 1970 – 1978 | 13.1% - 13.9% (HC)  13.9% - 17.6% (R) | 3.355.000 | Yes | EPF | No | Hospital category, Region | No | Yes |
| 1980 | Zdeb | Cesarean sections in upstate New York. 1968-1978 | 1968 – 1978 | 1.1% - 22.5% | 581.682 | No | Database | No | Hospital | No | No |
| 1981 | Lins | Cesarean section in four Rio de Janeiro Hospitals | 1977 – 1988 | 14.9% - 80.2% | 1.819 | No | EPF | No | Hospital | No | No |
| 1982 | Janowitz | Caesarean delivery in selected Latin American hospitals | 1977-1980 | 2.8% - 49.1% | 97.790 | No | EPF | No | Hospital | Yes | Yes |
| 1982 | Taylor | Caesarean section rates in New South Wales. 1977 | 1977 | 7.2% - 18.4% (H) 2.6% - 12.8% (R) | 77.996 | Yes | Database | No | Hospital. Region | Yes | Yes |
| 1983 | Adams | The use of obstetrical procedures in the care of low-risk women | 1977 – 1979 | 0% - 10.4% (H)  2.1% - 6.4% (HC) | 10.521 | No | Database | No | Hospital. Horpotal category | Yes | Yes |
| 1983 | Nilsen | A comparison of cesarean section frequencies in two Norwegian hospitals | 1974 – 1979 | 3.9% - 8.5% | 6.309 | No | Database | No | Hospital | No | No |
| 1983 | Placek | Cesarean section delivery rates: United States. 1981 | 1981 | 15.4% - 22.0% (HC)  15.9% - 20.0% (R) | 225.000 | Yes | Database | No | Hospital category. Region | No | No |
| 1983 | Williams | Controlling the rise in cesarean section rates by the dissemination of information from vital records | 1978 – 1980 | 0.0% - 24.1% (H) 10.5% - 12.2% (HC) | 1.051.717 | No | Database | Yes | Hospital. Hospital category | No | No |
| 1985 | Anderson | Explaining variations in cesarean section rates: patients. facilities or policies? | 1979 – 1982 | 16.5% - 21.0% (H) 17.1% - 20.2% (R) | 120.757 | No | Database | No | Hospital. Region | No | No |
| 1985 | Leveno | Cesarean section: an answer to the House of Horne | 1983 | 6.0% - 18.0% | 19.056 | No | Database | No | Hospital | Yes | No |
| 1986 | Nielsen | Caesarean section and perinatal mortality in Sweden in 1981 | 1981 | 8.0% - 19.0% (H)  12.4% - 12.5% (HC) | 93.678 | No | Database | No | Hospital. Hospital Category | Yes | No |
| 1987 | Notzon | Comparisons of national cesarean-section rates | 1970 – 1983 | 5.0% - 18.0% | ? | No | Other | No | National | No | No |
| 1987 | Shiono | Recent trends in cesarean birth and trial of labor rates in the United States | 1984 | 18.5% - 20.7% (HC)  17.1% - 20.4% (R) | 734.019 | Yes | Questionnaire | No | Hospital category.  Region | No | No |
| 1988 | Acker | Cesarean birth rate: small-geographic-area analysis | 1982 – 1985 | 13.5% - 30.4% | ? | No | Database | Yes | Region | No | No |
| 1988 | Rodrigues | Urban hospital cesarean section delivery rates in Paraiba State. Brazil. 1977-81 | 1977 – 1981 | 25.7% - 30.5% | 33.135 | No | Database | No | Hospital category | No | Yes |
| 1989 | Goyert | The physician factor in cesarean birth rates | 1986 – 1987 | 19.1% - 42.3% | 1.533 | No | EPF | Yes | Individual level | Yes | Yes |
| 1989 | Jonas | Caesarean section in South Australia. 1986 | 1986 | 13.0% - 22.1% (HC)  18.0% - 20.0% (R) | 19.800 | No | Database | No | Hospital category. Region | No | No |
| 1989 | Thiery | Frequency of cesarean deliveries in Belgium | 1985 | 7.1% - 8.7% | 2.896 | No | Other | No | Hospital | No | No |
| 1990 | Baruffi | Investigation of institutional differences in primary cesarean birth rates | 1977 – 1978 | 4.3% - 13.7% | 1.600 | Yes | EPF | Yes | Hospital | No | No |
| 1990 | DeMott | The Green Bay cesarean section study. I. The physician factor as a determinant of cesarean birth rates | 1986 – 1988 | 5.6% - 19.7% | 7.335 | No | Database | No | Individual | Yes | No |
| 1990 | Notzon | International differences in the use of obstetric interventions | 1975-1986 | 7.0% - 32.0% | ? | Both | Other | No | National | Yes | No |
| 1990 | Stewart | Diagnosis of dystocia and management with cesarean section among primiparous women in Ottawa-Carleton | 1984 | 11.8% - 19.6% | 3.887 | No | EPF | No | Hospital | No | No |
| 1991 | Notzon | International collaboratice effort (ICE) on birth weight. plurality. perinatal. and infant mortality. | 1970 – 1985 | 12.0% - 22.8% | ? | Yes | Other | No | National | No | Yes |
| 1991 | Oleske | The cesarean birth rate: influence of hospital teaching status | 1986 | 20.4% - 23.1% | 130.249 | No | Database | No | Hospital category | No | No |
| 1991 | Renwick | Caesarean section rates. Australia 1986: variations at state and small area level | 1986 | 15.1% - 26.3% | ? | No | Database | No | Region | No | No |
| 1991 | Saunders | Cesarean sections in Alberta from April 1979 to March 1988 | 1979 – 1988 | 10.3% - 22.3% | 128.562 | No | Database | No | Region | No | No |
| 1992 | Bertollini | Cesarean section rates in Italy by hospital payment mode: an analysis based on birth certificates | 1985 – 1987 | 21.3% – 34.7% | 136.666 | No | Database | No | Hospital category | No | No |
| 1992 | DeMott | The Green Bay cesarean section study. II. The physician factor as a determinant of cesarean birth rates for failed labor | 1986 – 1989 | 6.0% - 19.5% | 2.841 | No | EPF | No | Individual | Yes | No |
| 1992 | Kirsop | The influence of maternal age on Caesarean section rates | 1989 – 1990 | 18.3% - 22.5% | 6.880 | No | Database | No | Hospital | No | No |
| 1992 | Parazzini | Determinants of caesarean section rates in Italy | 1980 – 1983 | 11.8% - 13.3% (HC)  10.5% - 15.1% (R) | 322.363 | No | Database | No | Hospital category. Region | No | No |
| 1993 | De Muylder | Caesarean section rates in an African country | 1985 – 1986 | 2.2% - 16.8% | 19.363 | No | Other | No | Hospital | Yes | No |
| 1993 | McKenzie | Variation in cesarean section rates among hospitals in Washington State | 1987 | 17.5% - 36.0% | 69.578 | No | Database | No | Hospital category | No | No |
| 1993 | Rock | Variability and consistency of rates of primary and repeat cesarean sections among hospitals in two states | 1983 – 1988 | 3.8% - 33.3% (H) 9.2% - 10.8% (R) | ? | No | Database | No | Hospital. Region | No | No |
| 1993 | Stephenson | Patterns of use of obstetrical interventions in 12 countries | 1983 – 1988 | 6.5% - 19.5% | ? | No | Other | No | National | No | No |
| 1994 | Davis | Cesarean section rates in low-risk private patients managed by certified nurse-midwives and obstetricians | 1987 – 1990 | 8.5% - 12.8% | 8.795 | No | Database | No | Group of physicians | Yes | No |
| 1994 | Hendriksen | Cesarean section in twin pregnancies in two Danish counties with different cesarean section rates | 1989 | 28.0% - 57.0% | ? | No | Database | No | Region | Yes | No |
| 1994 | Notzon | Cesarean section delivery in the 1980s: international comparison by indication | 1980-1990 | 10.7%% - 23.6% | 416.195 | Both | Database | No | National | No | No |
| 1994 | Sanchez-Ramos | Cesarean section rates in teaching hospitals: a national survey | 1990 | 7.3% - 48.3% (H)  18.7% - 23.3% (R) | 945.707 | No | Questionnaire | No | Hospital. Region | No | Yes |
| 1994 | Sandmire | The Green Bay cesarean section study. III. Falling cesarean birth rates without a formal curtailment program | 1986 – 1992 | 5.9% - 14.4% | 5.655 | No | EPF | No | Individual | Yes | No |
| 1994 | Sperling | Indications for cesarean section in singleton pregnancies in two Danish counties with different cesarean section rates | 1989 | 8.3% - 15.2% | 11.341 | No | Database | No | Region | Yes | No |
| 1995 | Acien | Breech presentation in Spain. 1992: a collaborative study | 1992 | 13.6% - 17.2% | 102.038 | Yes | Questionnaire | No | Region | Yes | No |
| 1995 | Clarke | Changes in cesarean delivery in the United States. 1988 and 1993 | 1988 – 1993 | 20.5% - 29.0% (HC)  20.8% - 25.9% (R) | ? | Yes | Database | No | Hospital category. Region | No | No |
| 1995 | Heres | The Dutch obstetric intervention study--variations in practice patterns | 1990 | 5.8% - 22.7% | 28.934 | Yes | Database | No | Hospital | No | No |
| 1995 | Hueston | Site-to-site variation in the factors affecting cesarean section rates | 1990 – 1991 | 9.0% - 16.0% (GP)  11.0% - 19.0% (H) | 7.367 | Yes | EPF | Yes | Group of physicians. Hospital | No | Yes |
| 1995 | Johnson | Variation in caesarean and instrumental delivery rates in New Zealand hospitals | 1988 – 1993 | 9.6% - 20.8% | ? | No | Database | No | Hospital | No | No |
| 1995 | Signorelli | Risk factors for caesarean section in Italy: results of a multicentre study | 1991 – 1992 | 15.7% - 29.4% | 1.316 | Yes | Other | No | Hospital | No | No |
| 1995 | Turan | Cesarean section rates and perinatal outcomes in resident and midwife attended low risk deliveries | 1994 | 3.1% - 9.9% (GP)  3.1% - 9.9% (H) | 1.668 | No | Other | No | Group of physicians. Hospital | Yes | No |
| 1996 | Di Lallo | Cesarean section rates by type of maternity unit and level of obstetric care: an area-based study in central Italy | 1988 – 1989 | 22.5% - 42.0% | 90.338 | No | Database | No | Hospital category | No | No |
| 1996 | Hueston | Variations in cesarean section delivery for fetal distress | 1991 – 1992 | 0.9% - 3.0% | 6.440 | Yes | EPD | Yes | Hospital | No | No |
| 1996 | Millar | Declining cesarean section rates: a continuing trend? | 1979 – 1993 | 15.1% - 22.3% | 386.309 | No | Database | No | Region | No | No |
| 1997 | Elliott | The labor-adjusted cesarean section rate--a more informative method than the cesarean section "rate" for assessing a practitioner's labor and delivery skills | 1994 | 4.2% - 51.0% (I)  17.2% - 36.4% (GP) | 6.062 | No | Other | Yes | Individual. Group of physicians | No | No |
| 1997 | Menticoglou | Differences among obstetricians in caesarean section rates | 1989 – 1994 | 5.5% - 20.1% (I)  12.6% - 15.9% (GP) | 5.559 | No | Other | No | Individual. Group of physicians | Yes | No |
| 1997 | Murray | Cesarean birth trends in Chile. 1986 to 1994 | 1986 – 1994 | 20.5% - 37.2% | ? | No | Database | No | Region | No | No |
| 1998 | Aron | Impact of risk-adjusting cesarean delivery rates when reporting hospital performance | 1993 – 1995 | 6.3% - 26.5% | 26.127 | No | EPF | Yes | Hospital | No | No |
| 1998 | Chacham | The incidence of caesarean deliveries in Belo Horizonte. Brazil: Social and economic determinants | 1994 | 34.9% - 67.8% | 41.404 | No | Database | No | Hospital category | No | No |
| 1998 | Clark | Institutional influences on the primary cesarean section rate in Utah. 1992 to 1995 | 1992 – 1995 | 6.0% - 27.0% (H)  9.8% - 12.8% (HC) | 147.191 | No | Database | No | Hospital. Hospital category | No | No |
| 1998 | Rabilloud | Study of the variations of the cesarean sections rate in the Rhone-Alpes region (France): effect of women and maternity service characteristics | 1990 | 2.0% - 19.0% | 8.470 | Yes | Database | Yes | Hospital | No | No |
| 1999 | Belizan | Rates and implications of caesarean sections in Latin America: ecological study | 1993 – 1997 | 1.6% - 40.0% | ? | Yes | Other | No | National | Yes | Yes |
| 1999 | Glantz | Cesarean delivery risk adjustment for regional interhospital comparisons | 1998 | 17.1% - 39.2% | 6.798 | No | Database | Yes | Hospital | No | No |
| 1999 | Gregory | Cesarean deliveries for medicaid patients: a comparison in public and private hospitals in Los Angeles county | 1991 | 13.2% - 24.5% | 92.798 | No | Database | No | Hospital category | No | No |
| 1999 | Main | Reducing cesarean birth rates with data-driven quality improvement activities | 1994 | 9.5% - 33.3% (I)  20.7% - 25.1% (H) | ? | No | Database | No | Individual. Hospital | Yes | No |
| 1999 | Menard | Cesarean delivery rates in the United States. The 1990s | 1996 | 15.1% - 26.6% | ? | No | Database | No | Region | No | No |
| 1999 | Onion | Primary cesarean section rates in uninsured. Medicaid and insured populations of predominantly rural northern New England | 1990 – 1992 | 14.1% - 15.4% | 101.655 | No | Database | Yes | Region | No | No |
| 1999 | Poma | Effects of obstetrician characteristics on cesarean delivery rates. A community hospital experience | 1994 – 1997 | 5.6% - 25.2% | 7.827 | No | EPF | No | Individual | Yes | Yes |
| 2000 | Beilin | The effect of the obstetrician group epidural analgesia on the risk for cesarean delivery in nulliparous women | 1997 – 1999 | 8.0% - 34.0% | 1.228 | No | Database | No | Group of physicians | No | Yes |
| 2000 | Chanrachakul | Epidemic of cesarean section at the general. private and university hospitals in Thailand | 1998 | 22.6% - 48.1% | 1.073.403 | Yes | Questionnaire | Yes | Hospital category | No | Yes |
| 2000 | Librero | Inter-hospital variations in caesarean sections. A risk adjusted comparison in the Valencia public hospitals | 1994 – 1995 | 14.6% - 24.4% | 36.819 | No | Database | Yes | Hospital | No | No |
| 2000 | Roberts | Rates for obstetric intervention among private and public patients in Australia: population based descriptive study | 1996 – 1997 | 13.8% - 16.4% | 170.706 | No | Database | Yes | Hospital category | Yes | No |
| 2000 | Whitsel | Adjustment for case mix in comparisons of cesarean delivery rates: university versus community hospitals in Vermont | 1997 – 1998 | 8.5% - 11.4% | 5.705 | No | Database | Yes | Hospital | No | No |
| 2001 | Gichangi | Rate of caesarean section as a process indicator of safe-motherhood programmes: the case of Kenya | 1994 – 1998 | 0.3% - 37.7% (H)  0.1% - 4.0% (R) | 815.890 | Yes | Other | No | Hospital. Region | No | No |
| 2001 | Gonzalez-Perez | Caesarean sections in Mexico: are there too many? | 1983 – 1998 | 22.6% - 48.1% | 1.706.659 | No | Database | No | Hospital category | No | No |
| 2001 | Gregory | Variation in elective primary cesarean delivery by patient and hospital factors | 1995 | 3.4% - 4.5% | 463.196 | No | Database | No | Hospital category | No | No |
| 2001 | Janssen | Differences in institutional cesarean delivery rates-the role of pain management | ? | 6.7% - 20.7% | 857 | Yes | EPD | Yes | Hospital | Yes | No |
| 2001 | Leung | Rates of cesarean births in Hong Kong: 1987-1999 | 1987 – 1999 | 19.3% – 49.9% | ? | Yes | Other | No | Hospital category | No | No |
| 2001 | Menacker | Trends in cesarean birth and vaginal birth after previous cesarean. 1991-99 | 1991 – 1999 | 14.4% - 37.8% | 3.959.417 | No | Database | No | Region | No | No |
| 2001 | Rabilloud | Maternity hospitals ranking on prophylactic caesarean section rates: uncertainty associated with ranks | 1990 | 2.0% - 19.0% | 8.470 | Yes | Other | Yes | Hospital | No | No |
| 2001 | Spetz | Physician incentives and the timing of cesarean sections: evidence from California | 1995 | 15.8% - 24.2% | 552.393 | No | Database | No | Hospital category | No | No |
| 2001 | Zezai | Caesarean section rate as a process indicator of safe motherhood programmes: the case of Midlands Province | 1999 | 0.5% - 34.5% (H)  8.0% - 24.0% (HC)  0.2% - 7.1% (R) | 3.316.382 | Yes | Other | No | Hospital. Hospital category. Region | Yes | Yes |
| 2002 | Kambo | A critical appraisal of cesarean section rates at teaching hospitals in India | 1993 – 1999 | 9.1% - 54.0% | 7.017 | Yes | Database | No | Hospital | No | No |
| 2002 | Mishra | Delivery-related complications and determinants of caesarean section rates in India | 1992 – 1993 | 15.3% - 64.0% | ? | Yes | Questionnaire | Yes | Region | No | Yes |
| 2003 | Allen | Assisted delivery in the teenage population: the effect of inter-hospital variation. deprivation. and age | 1994 – 1997 | 6.0% - 12.5% | 10.514 | No | Database | Yes | Hospital | No | Yes |
| 2003 | Ravindran | Caesarean section rates in government hospitals in Malaysia. 2000-2001 | 2000 – 2001 | 7.4% - 22.3% | 361.206 | No | Database | No | Region | No | No |
| 2003 | Sreevidya | High caesarean rates in Madras (India): a population-based cross sectional study | 1997 – 1999 | 19.8% - 46.7% | 780 | Yes | Questionnaire | Yes | Hospital category | No | No |
| 2004 | Bailit | Stability of risk-adjusted primary cesarean delivery rates over time | 1995 – 1998 | 3.0% - 23.0% | 54.425 | No | Database | Yes | Hospital | No | No |
| 2004 | Jurdi | Caesarean section rates in the Arab region: a cross-national study | 1993 – 2001 | 1.4% - 16.0% | ? | ? | Other | No | National | Yes | Yes |
| 2004 | Khawaja | Rising trends in cesarean section rates in Egypt | 1989 – 2000 | 20.8% - 23.2% | 11.361 | Yes | Questionnaire | No | Hospital category | No | No |
| 2004 | Khawaja | Determinants of caesarean section in Egypt: evidence from the demographic and health survey | 2000 | 20.1%- 23.5% (HC)  14.8% - 26.2% (R) | 4.032 | Yes | Questionnaire | Yes | Hospital category. Region | No | No |
| 2004 | Lin | Institutional factors in cesarean delivery rates: policy and research implications | 2000 | 31.8% - 35.1% (HC)  28.0% - 35.6% (R) | 270.774 | No | Database | Yes | Hospital category. Region | No | Yes |
| 2004 | Linton | Effect of managed care enrollment on primary and repeat cesarean rates among U.S. Department of Defense health care beneficiaries in military and civilian hospitals worldwide. 1999-2002 | 1999 – 2002 | 20.1% - 21.4% | 365.648 | No | Database | No | Hospital category | No | Yes |
| 2004 | Liu | Recent trends in caesarean delivery rates and indications for caesarean delivery in Canada | 1994 – 2001 | 12.0% - 25.8% | 240.643 | No | Database | No | Region | No | No |
| 2005 | Capon | Case-mix adjusted odds ratios as an alternative way to compare hospital performances | 2001 | 14.8% - 66.4% | 41.755 | No | Database | Yes | Hospital | No | No |
| 2005 | Fischer | Differences between hospitals in cesarean rates for term primigravidas with cephalic presentation | 1998 – 2000 | 16.6% - 20.3% | 324 | Yes | EPF | No | Hospital | Yes | Yes |
| 2005 | Griffiths | A prospective observational study of emergency caesarean section rates and the effect of the labour ward experience | 2003 – 2004 | 10.3% - 25.5% | 817 | No | Database | No | Group of physicians | No | Yes |
| 2005 | Korst | Rethinking the cesarean rate: how pregnancy complications may affect interhospital comparisons | 1995 – 1995 | 8.8% - 12.8% | 443.532 | No | Database | No | Hospital category | No | No |
| 2005 | Linton | Clinical case mix adjustment of cesarean delivery rates in U.S. military hospitals. 2002 | 2002 | 20.9% - 23.7% (HC)  19.7% - 23.4% (R) | 53.215 | No | Database | Yes | Hospital category. Region | No | No |
| 2005 | Mossialos | An investigation of Caesarean sections in three Greek hospitals: the impact of financial incentives and convenience | 2002 | 41.6% - 53.0% | 376 | No | EPF | Yes | Hospital category | No | No |
| 2005 | Paranjothy | How much variation in CS rates can be explained by case mix differences? | 2000 | 6.0% - 66.0% | 147.087 | No | Database | Yes | Hospital | No | No |
| 2005 | Zeteroglu | Cesarean delivery rates in adolescent pregnancy | 1999 – 2003 | 24.7% - 27.9% | 40.391 | No | Other | No | Hospital | No | No |
| 2006 |  | Rates of cesarean delivery among Puerto Rican women--Puerto Rico and the U.S. mainland. 1992-2002 | 1992 – 2002 | 21.9% - 31.4% | 31.719 | No | Database | No | Region | No | No |
| 2006 | Baicker | Geographic variation in the appropriate use of cesarean delivery | 1995 – 1998 | 13.4% - 26.0% | 10.161.953 | No | Database | Yes | Region | No | Yes |
| 2006 | Hsu | Risk adjustment for inter-hospital comparisons of caesarean section rates in Taipei municipal hospitals | 1999 – 2001 | 26.3% - 38.4% | 27.693 | No | Database | Yes | Hospital | No | No |
| 2006 | Main | Is there a useful cesarean birth measure? Assessment of the nulliparous term singleton vertex cesarean birth rate as a tool for obstetric quality improvement | 2001 – 2003 | 10.5% - 30.2% | 41.416 | No | Database | Yes | Hospital | No | No |
| 2006 | Ronsmans | Socioeconomic differentials in caesarean rates in developing countries: a retrospective analysis | 1988 – 2002 | 0.3% - 36.4% | 199.916 | Yes | Questionnaire | No | National | No | No |
| 2006 | Stanton | Levels and trends in cesarean birth in the developing world | 1990 – 2003 | 0.4% - 40.0% | ? | Yes | Other | No | Hospital | No | No |
| 2006 | Villar | Caesarean delivery rates and pregnancy outcomes: the 2005 WHO global survey on maternal and perinatal health in Latin America | 2004 – 2005 | 28.0% - 75.0%(HC)^1^  28.0% - 75.0% (N)^[[1]](#footnote-1)^ | 97095 | Yes | Questionnaire | No | Hospital category. National | No | No |
| 2006 | Wanyonyi | Caesarian section rates and perinatal outcome at the Aga Khan University Hospital. Nairobi | 2001 – 2004 | 34.5% - 41.7% | 2.142 | No | EPF | No | Group of Physicians | No | No |
| 2006 | Xirasagar | Do group practices have lower caesarean rates than solo practice obstetric clinics? Evidence from Taiwan | 2000 – 2002 | 31.7% - 34.1% | 253.618 | No | Database | No | Group of physicians | No | Yes |
| 2007 | Betran | Rates of caesarean section: analysis of global. regional and national estimates | ? | 0.4% - 40.5% | ? | Both | Other | No | National | Yes | No |
| 2007 | Clark | Variation in the rates of operative delivery in the United States | 2004 | 9.0% - 37.0% (H)  18.0% - 20.0% (HC)  15.5% - 21.0% (R) | 219.168 | No | Database | No | Hospital. Hospital category. Region | No | No |
| 2007 | Hong | Factors related to the high cesarean section rate and their effects on the "price transparency policy" in Beijing. China | 2002 | 54.8% - 62.0% | 680 | Yes | Other | No | Hospital | No | Yes |
| 2007 | Hsu | Cesarean births in Taiwan | 1998 – 2000 | 32.2% - 34.2% | 297.865 | No | Database | No | Hospital category | No | No |
| 2007 | Kilsztajn | Caesarean sections and maternal mortality in Sao Paulo | 2003 | 32.9% - 80.4% | 606.049 | No | Database | No | Hospital category | No | No |
| 2007 | Lee | Effects of the DRG-based prospective payment system operated by the voluntarily participating providers on the cesarean section rates in Korea | 2003 | 36.8% - 42.9% | 179.222 | Yes | Database | Yes | Hospital category | No | Yes |
| 2007 | Ribeiro | Why are the rates of cesarean section in Brazil higher in more developed cities than in less developed ones? | 1994 – 1998 | 33.7% - 50.8% | 5.289 | Yes | Questionnaire | Yes | Region | No | Yes |
| 2007 | Sufang | Delivery settings and caesarean section rates in China | 1993 – 2002 | 12.5% - 24.7% | 716 | Yes | Questionnaire | No | Region | No | No |
| 2007 | Tamim | Incidence and correlates of cesarean section in a capital city of a middle-income country | 2001 – 2002 | 19.3% - 31.1% | 18.837 | No | Other | No | Hospital category | No | No |
| 2007 | Tamim | Cesarean delivery among nulliparous women in Beirut: assessing predictors in nine hospitals | 2001 – 2002 | 12.5% - 31.4% | 6.668 | No | Other | Yes | Hospital category | Yes | Yes |
| 2008 | Aelvoet | Screening for inter-hospital differences in cesarean section rates in low-risk deliveries using administrative data: an initiative to improve the quality of care | 2001 – 2004 | 8.8% - 19.3% | 332.411 | No | Other | Yes | Hospital | Yes | Yes |
| 2008 | Carayol | Determinants of caesarean section in Lebanon: geographical differences | 1999 – 2000 | 7.6% - 13.4% | 3.850 | Yes | Questionnaire | Yes | Region | No | Yes |
| 2008 | Chauhan | Primary cesarean delivery among uncomplicated term nulliparous parturients: the influence of group practice within a community hospital | 2002 – 2003 | 15.0% - 32.0% | 1.217 | No | Database | Yes | Group of physicians | Yes | Yes |
| 2008 | Coonrod | Nulliparous term singleton vertex cesarean delivery rates: institutional and individual level predictors | 2005 | 10.3% - 34.2% | 28.863 | No | Database | Yes | Hospital | No | Yes |
| 2008 | De Almeida | Significant differences in cesarean section rates between a private and a public hospital in Brazil | 1999 | 18.9% - 84.3% | 5.800 | No | EPF | No | Hospital | No | Yes |
| 2008 | Grewal | Primary cesarean delivery among parous women in the united states. 1990-2003 | 1990 – 2003 | 4.0% - 17.8% | 1.818.603 | No | Database | No | Region | No | No |
| 2008 | Lee | Population trends in cesarean delivery for breech presentation in the United States. 1997-2003 | 1997 – 2003 | 62.0% - 97.0% | 1.067.989 | No | Database | No | Hospital | No | No |
| 2008 | Lerchl | Where are the Sunday babies? III. Caesarean sections. decreased weekend births. and midwife involvement in Germany | 2003 | 19.2% - 30.5% | 706.721 | No | Database | No | Region | No | No |
| 2009 | Ahmad-Nia | Caesarean section in the Islamic Republic of Iran: prevalence and some sociodemographic correlates | 1998 – 2000 | 6.1% - 50.3% | 17.991 | Yes | Questionnaire | No | Region | No | No |
| 2009 | Ba'aqeel | Cesarean delivery rates in Saudi Arabia: a ten-year review | 1997 – 2006 | 10.0% - 33.0% (H)  18.0% - 21.0% (HC) | ? | No | Database | No | Hospital. Hospital category | No | No |
| 2009 | Brennan | Comparative analysis of international cesarean delivery rates using 10-group classification identifies significant variation in spontaneous labor | 2005 – 2006 | 15.7% - 34.0% | 47.402 | No | Database | No | Hospital | No | No |
| 2009 | Festin | Caesarean section in four South East Asian countries: reasons for. rates. associated care practices and health outcomes | 2005 | 12.0% - 39.0% (H)  19.1% - 34.8% (N) | 9.550 | Yes | EPF | No | Hospital. National | Yes | Yes |
| 2009 | Howell | Trends and determinants of caesarean sections births in Queensland. 1997-2006 | 2006 | 26.9% - 48.0% | 55.719 | No | Database | No | Hospital category | No | No |
| 2009 | Lipkind | Disparities in cesarean delivery rates and associated adverse neonatal outcomes in New York City hospitals | 1996 – 2003 | 20.5% - 21.2% | 321.308 | No | Database | Yes | Hospital category | Yes | No |
| 2009 | Taljaard | Understanding the factors associated with differences in caesarean section rates at hospital level: the case of Latin America | 2004 – 2005 | 0.0% - 85.0% (H)  24.2% - 41.4% (N) | 97.095 | No | Other | Yes | Hospital. National | No | Yes |
| 2009 | Triunfo | The effect of physicians' remuneration system on the Caesarean section rate: the Uruguayan case | 2003 | 20.0% - 38.9% (HC)  16.0% - 40.0% (N) | 158.635 | No | Database | Yes | Hospital category. National | No | No |
| 2010 | Bogg | Dramatic increase of Cesarean deliveries in the midst of health reforms in rural China | 2004 – 2007 | 18.6% - 60.0% | 26.125 | No | Other | No | Region | No | Yes |
| 2010 | Bragg | Variation in rates of caesarean section among English NHS trusts after accounting for maternal and clinical risk: cross sectional study | 2008 | 13.6% - 31.9% | 620.604 | No | Database | Yes | Hospital | No | No |
| 2010 | Hanley | Regional variation in the cesarean delivery and assisted vaginal delivery rates | 2004 – 2007 | 16.1% - 27.5% | 116.839 | No | Database | Yes | Region | No | No |
| 2010 | Karlström | Caesarean section without medical reason 1997- 2006: A swedish register study | 1997 – 2006 | 14.1% - 16.7% | 301.648 | No | Database | Yes | Region | No | No |
| 2010 | Klemetti | Cesarean section delivery among primiparous women in rural China: an emerging epidemic | 1991 – 2002 | 8.3% - 26.6% (HC)  5.0% - 19.0% (R) | 3.050 | Yes | Questionnaire | No | Hospital category. Region | No | No |
| 2010 | Lumbiganon | Method of delivery and pregnancy outcomes in Asia: the WHO global survey on maternal and perinatal health 2007-08 | 2007 – 2008 | 14.7% - 46.2% | 107.950 | Yes | EPF | No | National | No | No |
| 2010 | Mendoza-Sassi | Risk factors for cesarean section by category of health service | 2007 | 42.6% - 85.8% | 2.557 | No | Questionnaire | No | Hospital category | No | No |
| 2010 | Zeitlin | Variability in caesarean section rates for very preterm births at 28-31 weeks of gestation in 10 European regions: results of the MOSAIC project | 2002 – 2003 | 13.9% - 38.3% | 533.650 | No | EPF | No | Region | Yes | No |
| 2011 | Declercq | Is a Rising Cesarean Delivery Rate Inevitable? Trends in Industrialized Countries. 1987 to 2007 | 1987 – 2007 | 7.0% - 39.8% | ? | ? | Other | No | National | No | No |
| 2011 | Fesseha | A national review of cesarean delivery in Ethiopia | 2007 – 2008 | 15.0% - 46.1% | 174.561 | Yes | Other | No | Hospital category | Yes | No |
| 2011 | Jurgens | Clients' and providers' perspectives on caesarean sections: An operational study into the high caesarean section rate in Georgia | ? | 10.0% - 77.0% | ? | Yes | Questionnaire | No | Region | No | No |
| 2011 | MacDorman | Recent trends and patterns in cesarean and vaginal birth after cesarean (VBAC) deliveries in the United States | 1990 – 2008 | 23.8% - 37.2% | ? | No | Database | No | Region | No | No |
| 2011 | Snyder | The influence of hospital type on induction of labor and mode of delivery | 2006 – 2007 | 25.7% - 26.1% | 283.370 | No | Database | No | Hospital category | No | No |
| 2011 | Stivanello | Risk adjustment for inter-hospital comparison of caesarean delivery rates in low-risk deliveries | 2007 – 2009 | 13.2% - 40.2% | 87.849 | No | Database | Yes | Hospital | No | No |
| 2011 | Turner | The use of quality control performance charts to analyze cesarean delivery rates nationally | 2009 | 18.7% - 35.6% | 74.278 | No | Database | No | Hospital | No | No |
| 2011 | Zizza | Caesarean section in the world: a new ecological approach | ? | 0.4% - 42.3% | ? | Both | Other | No | National | Yes | No |
| 2012 | Briand | Individual and institutional determinants of caesarean section in referral hospitals in Senegal and Mali: a cross-sectional epidemiological survey | 2007 – 2008 | 8.0% - 46.0% | 86.505 | No | Database | No | Hospital | No | Yes |
| 2012 | Chu | Cesarean section rates and indications in sub-Saharan Africa: a multi-country study from Medecins sans Frontieres | 2010 – 2011 | 4.1% - 16.8% | 20.080 | No | Database | No | Hospital | Yes | No |
| 2012 | Huang | Impact of alternative reimbursement strategies in the new cooperative medical scheme on caesarean delivery rates: a mixed-method study in rural China | 2005 – 2008 | 58.2% - 84.3% | 1.718 | Yes | Questionnaire | Yes | Region | No | Yes |
| 2012 | Lutomski | Regional variation in obstetrical intervention for hospital birth in the Republic of Ireland. 2005-2009 | 2005 – 2009 | 22.8% - 29.0% | 323.588 | No | Database | Yes | Region | No | No |
| 2012 | Miller | Going public: do risk and choice explain differences in caesarean birth rates between public and private places of birth in Australia? | 2009 | 27.4% - 42.6% | 757 | Yes | Questionnaire | No | Hospital category | No | No |
| 2012 | Qin | Clinical indications and determinants of the rise of cesarean section in three hospitals in rural China | 1997 – 2003 | 54.0% - 65.0% | 905 | No | EPF | No | Hospital | No | No |
| 2013 | Bahadori | The trend of caesarean delivery in the Islamic Republic of Iran | 2005 – 2009 | 36.4% - 64.3% (HC). 22.4% - 74.3% (R) | ? | ? | Other | No | Hospital category. Region | No | No |
| 2013 | Caceres | Hospital differences in cesarean deliveries in Massachusetts (US) 2004-2006: the case against case-mix artifact | 2004 – 2006 | 14.9% - 36.4% | 80.265 | No | Database | Yes | Hospital | No | No |
| 2013 | Cavallaro | Trends in caesarean delivery by country and wealth quintile: cross-sectional surveys in southern Asia and sub-Saharan Africa. | 1985 – 2011 | 0.4% - 8.4% | 686.789 | Yes | Questionnaire | No | National | No | No |
| 2013 | Di Mario | Baby-friendly hospitals and cesarean section rate: a survey of Italian hospitals | 2009 | 24.9% - 30.9% (HC). 23.7% - 62.0% (R) | 596.694 | No | Database | No | Hospital category. Region | No | Yes |
| 2013 | Einarsdottir | Role of public and private funding in the rising caesarean section rate: a cohort study | 1996 – 2008 | ?^[[2]](#footnote-2)^ | 155.646 | No | Database | No | Hospital category | No | No |
| 2013 | Gonzales | Pregnancy outcomes associated with Cesarean deliveries in Peruvian public health facilities | 2000 – 2010 | 20.6% - 32.8% | 563.668 | No | Database | No | Region | Yes | No |
| 2013 | Huang | A mixed-method study of factors associated with differences in caesarean section rates at community level: the case of rural China | 2005 – 2006 | 46.0% - 64.7% | 2.326 | No | Questionnaire | No | Region | No | Yes |
| 2013 | Kelly | Examining caesarean section rates in Canada using the Robson classification system | 2007 – 2013 | 27.0% - 32.0% | 240.225 | No | Database | No | Region | No | No |
| 2013 | Kozhimannil | Cesarean delivery rates vary tenfold among us hospitals; Reducing variation may address quality and cost issues | 2009 | 7.1% - 69.9% (H)  32.0% - 33.4% (HC) | 817.318 | No | Database | No | Hospital. Hospital category | No | No |
| 2013 | Kyvernitakis | Rising cesarean rates of twin deliveries in Germany from 1990 to 2012 | 1990 – 2012 | 66.0% - 75.8% | 867 | No | Database | No | Hospital category | No | No |
| 2013 | Lee | Unexplained variation in hospital caesarean section rates | 2009 – 2010 | 11.8% - 47.4% | 183.310 | No | Database | No | Hospital | No | No |
| 2013 | Maso | Interinstitutional variation of caesarean delivery rates according to indications in selected obstetric populations: a prospective multicenter study | ? | 14.3% - 34.1% | 15.726 | No | Database | No | Hospital | Yes | No |
| 2013 | Mikolajczyk | Regional variation in caesarean deliveries in Germany and its causes | 2004 – 2006 | 19.8% - 34.4% | 95.486 | No | Database | No | Region | No | No |
| 2013 | Pallasmaa | Variation in cesarean section rates is not related to maternal and neonatal outcomes | 2005 | 12.9% - 25.1% | 19.764 | No | Database | Yes | Hospital | Yes | No |
| 2013 | Sudhof | Local use of geographic information systems to improve data utilisation and health services: mapping caesarean section coverage in rural Rwanda | 2009 | 6.8% - 18.4% | 5.988 | Yes | Database | No | Region | Yes | No |
| 2013 | Teixeira | Risk of caesarean section after induced labour: do hospitals make a difference? | 2005 – 2006 | 32.5% - 48.4% | 2.041 | Yes | Other | No | Hospital | No | No |
| 2014 | Al Rifai | Rising cesarean deliveries among apparently low-risk mothers at university teaching hospitals in Jordan: analysis of population survey data. 2002-2012 | 2002 – 2012 | 29.4% - 32.2% (HC)  28.2% - 31.2% (R) | 6.365 | Yes | Questionnaire | No | Hospital category. Region | No | No |
| 2014 | Bahadori | The trend of caesarean delivery in the Islamic Republic of Iran | 2005 – 2009 | 22.4% - 74.3% | ? | Both | Other | No | Region | No | No |
| 2014 | Biro | Is place of birth associated with mode of birth? The effect of hospital on caesarean section rates in a public metropolitan health service | 2010 – 2011 | 12.0% - 17.6% | 2.736 | No | Database | Yes | Hospital | No | No |
| 2014 | Brown | Caesarean section rates in Southwestern Ontario: changes over time after adjusting for important medical and social characteristics | 1999 – 2010 | 21.1% - 26.6% | 23.192 | No | Database | Yes | Hospital | No | No |
| 2014 | Da Silva Campi | Spatial distribution of C-sections within the state of São Paulo | 2003 – 2007 | 31.7% - 78.8% | 3.045.293 | No | Database | No | Region | No | No |
| 2014 | Escuriet | Obstetric interventions in two groups of hospitals in Catalonia: a cross-sectional study | 2011 | 19.8% - 42.3% | 78.570 | No | Database | No | Hospital category | No | No |
| 2014 | Ganchimeg | Mode and timing of twin delivery and perinatal outcomes in low- and middle-income countries: a secondary analysis of the WHO Multicountry Survey on Maternal and Newborn Health | 2010 – 2011 | 11.7% - 88.3% | 2.013 | Yes | EPF | No | National | No | No |
| 2014 | Ghotbi | Women's knowledge and attitude towards mode of delivery and frequency of cesarean section on mother's request in six public and private hospitals in Tehran. Iran. 2012 | 2010 – 2011 | 78.5% - 91.9% | 600 | ? | Questionnaire | No | Hospital category | No | No |
| 2014 | Hopkins | The impact of payment source and hospital type on rising cesarean section rates in Brazil. 1998 to 2008 | 1997 – 2008 | 41.2% - 85.0% | 3.660 | Yes | Questionnaire | No | Hospital category | No | No |
| 2014 | Ishikawa | Cesarean delivery and perinatal mortality rates in Japan. 2007-2011 | 2007 – 2011 | 11.8% - 23.5% | 5.016.721 | No | Database | No | Region | Yes | No |
| 2014 | Kozhimannil | Maternal clinical diagnoses and hospital variation in the risk of cesarean delivery: analyses of a National US Hospital Discharge Database | 2009 – 2010 | 11.0% - 36.0% (H)  31.7% - 33.5% (HC) | 1.475.457 | ? | Database | Yes | Hospital. Hospital category | No | Yes |
| 2014 | Nathan | Differences in the average Caesarean section rate across levels of hospital care in Gauteng. South Africa | 2014 – 2017 | 33.3% - 55.5% (H)  18.0% - 42.0% (HC) | ? | ? | Other | No | Hospital. Hospital category | No | No |
| 2014 | Neuman | Prevalence and determinants of caesarean section in private and public health facilities in underserved South Asian communities: cross-sectional analysis of data from Bangladesh. India and Nepal | 2005 – 2012 | 21.0% - 90.0% | 45.268 | Yes | Other | Yes | Hospital | No | Yes |
| 2014 | Osterman | Trends in low-risk cesarean delivery in the United States. 1990-2013 | 1990 – 2013 | 16.7% - 33.1% | 1.269.636 | No | Database | No | Region | No | No |
| 2014 | Osterman | Primary cesarean delivery rates. by state: results from the revised birth certificate. 2006-2012 | 2006 – 2012 | 12.5% - 26.9% | ? | No | Database | No | Region | No | No |
| 2014 | Prosser | Why 'down under' is a cut above: A comparison of rates of and reasons for caesarean section in England and Australia | 2009 - 2010 | 25.1% - 36.2% | 8.717 | Yes | Questionnaire | Yes | National | No | No |
| 2014 | Rahman | Determinants of Caesarean Risk Factor in Northern Region of Bangladesh: A Multivariate Analysis | 2010 | 30.3% - 93.5% | 1.142 | Yes | Questionnaire | Yes | Hospital category | No | No |
| 2015 | Bannister-Tyrrell | Variation in hospital caesarean section rates for preterm births | 2007 – 2011 | 17.4% - 48.3% | 20.247 | No | Database | Yes | Hospital | Yes | Yes |
| 2015 | Barcaite | Cesarean section rates in Lithuania using Robson Ten Group Classification System | 2012 | 21.6% - 30.2% | 25.373 | ? | Other | No | Hospital category | No | No |
| 2015 | Barros | Cesarean sections in Brazil: will they ever stop increasing? | 2000 – 2011 | 9.5% - 91.2% | 2.900.000 | No | Database | No | Region | No | No |
| 2015 | Escuriet-Peiro | Impact of maternity care policy in Catalonia: a retrospective cross-sectional study of service delivery in public and private hospitals | 2007 – 2012 | 19.6% - 39.4% | 68.215 | No | Database | No | Hospital category | No | No |
| 2015 | García-Armesto | Potential of geographical variation analysis for realigning providers to value-based care. ECHO case study on lower-value indications of C-section in five European countries | 2002 – 2009 | 18.2% - 31.8% | 1.207.032 | No | Other | No | National | No | No |
| 2015 | Gross | Interinstitutional variations in mode of birth after a previous caesarean section: a cross-sectional study in six German hospitals. | 2011 | 26.7% - 34.1% (H)  22.2% - 34.0% (R) | 12.060 | No | Database | No | Hospital.  Region | No | No |
| 2015 | Irani | Challenges affecting access to cesarean delivery and strategies to overcome them in low-income countries | 2008 – 2011 | 1.5% - 7.1% | ? | ? | Other | No | National | Yes | Yes |
| 2015 | Islam | Rate of cesarean delivery at hospitals providing emergency obstetric care in Bangladesh | 2008 | 17.8% - 56.3% | 1.043 | No | Database | No | Hospital | No | No |
| 2015 | Long | Caesarean section rates in Mozambique | 1995 – 2011 | 2.3% - 8.3% | 6.256 | Yes | Questionnaire | Yes | Region | No | No |
| 2015 | McDonald | Cesarean birth in the border region: a descriptive analysis based on US Hispanic and Mexican birth certificates | 2009 | 22.5% - 51.1% | 3.000.000 | No | Datanase | No | Region | No | No |
| 2015 | Nippita | Variation in hospital caesarean section rates and obstetric outcomes among nulliparae at term: a population-based cohort study | 2009 – 2010 | 2.6% - 20.2% | 67.239 | No | Database | Yes | Hospital | Yes | Yes |
| 2015 | Schemann | Variation in hospital caesarean section rates for women with at least one previous caesarean section: A population based cohort study | 2007 – 2011 | 47.9% - 94.4% | 61.894 | No | Database | Yes | Hospital | Yes | Yes |
| 2015 | Vogel | Use of the Robson classification to assess caesarean section trends in 21 countries: a secondary analysis of two WHO multicountry surveys | 2004 – 2011 | 5.3% - 46.2% | 227.811 | Yes | Questionnaire | No | National | No | No |
| 2016 | Betrán | The increasing trend in caesarean section rates: Global. regional and national estimates: 1990-2014 | 1990 – 2014 | 1.4% - 56.4% | ? | Both | Other | No | National | No | No |
| 2016 | Brick | Recent Trends in Vaginal Birth After Caesarean Section | 1990 – 2012 | 24.9% - 35.2% | ? | ? | Other | No | Regional | No | No |
| 2016 | Freitas | Association between institutional factors. birth care profile. and cesarean section rates in Santa Catarina | 2012 | 45.1% - 89.0% | 61.278 | No | Database | Yes | Hospital category | No | No |
| 2016 | Macfarlane | Wide differences in mode of delivery within Europe: risk-stratified analyses of aggregated routine data from the Euro-Peristat study | 2010 | 14.8% - 52.2% | 4.170.000 | Both | Other | No | National | No | No |
| 2016 | Meda | Rate of and factors associated with indications for cesarean deliveries: Results of a national review in Burkina Faso | 2009 – 2010 | 0.8% - 4.5% | 697.103 | Yes | Questionnaire | No | Region | No | No |
| 2016 | Metz | Variation in primary cesarean delivery rates by individual physician within a single-hospital laborist model | 2007 – 2014 | 12.5% - 35.9% | 2.224 | No | EPF | No | Individual | Yes | No |
| 2016 | Mistry | Variation in the Rate of Cesarean Section Across U.S. Hospitals. 2013: Statistical Brief #211 | 2013 | 31.5% - 34.4% (HC)  29.8% - 34.9% (R) | 3.535.946 | No | Database | No | Hospital category. Region | No | No |
| 2016 | Nakamura-Pereira | Use of Robson classification to assess cesarean section rate in Brazil: The role of source of payment for childbirth | 2011 – 2012 | 42.9% - 87.9% | 23.894 | Yes | Questionnaire | No | Hospital category | No | No |
| 2016 | Ono | Comparative analysis of cesarean section rates using Robson Ten-Group Classification System and Lorenz curve in the main institutions in Japan | 2013 | 28.4% - 42.6% | 68.702 | Yes | Database | No | Hospital category | No | No |
| 2016 | Schemann | Variation in and factors associated with timing of low risk. pre-labour repeat caesarean sections in NSW. 2008-2011 | 2008 – 2011 | 6.1% - 76.6% | 15.163 | No | Database | Yes | Hospital | No | Yes |
| 2016 | Sebastiao | Hospital variation in cesarean delivery rates: contribution of individual and hospital factors in Florida | 2004 – 2012 | 7.4% - 48.8% (H)  22.5% - 26.4% (HC). 19.7% - 33.2% (R) | 412.192 | No | Database | Yes | Hospital. Hospital category. Region | No | Yes |
| 2016 | Sinnott | National Variation in Caesarean Section Rates: A Cross Sectional Study in Ireland | 2009 | 18.2% - 35.1% | 70.889 | No | Database | Yes | Hospital | No | No |
| 2017 | Alonso | Caesarean birth rates in public and privately funded hospitals: a cross-sectional study | 2011 – 2012 | 43.4% - 84.8% | 9.828 | Yes | Questionnaire | Yes | Hospital category | No | No |
| 2017 | Al Rifai | Trend of caesarean deliveries in Egypt and its associated factors: evidence from national surveys. 2005-2014 | 2005 – 2014 | 12.5% - 62.3% (HC)  0.9% - 39.0% (R) | 11.391 | Yes | Questionnaire | No | Hospital category. Region | No | No |
| 2017 | Edmonds | Variation in Cesarean Birth Rates by Labor and Delivery Nurses | 2013 – 2015 | 8.3% - 48.0% | 3.031 | No | EPF | No | Individual | Yes | No |
| 2017 | Guendelman | Prevalence. Disparities. And Determinants Of Primary Cesarean Births Among First-Time Mothers In Mexico | 2014 | 38.0% - 82.0% | 600.124 | No | Database | Yes | Hospital category | Yes | No |
| 2017 | Ishaq | Frequency and evaluation of the perceptions towards caesarean section among pregnant women attending public hospitals in Pakistan and the implications | 2014 - 2015 | 8.9% - 83.7% | 23.236 | Yes | Questionnaire | No | Hospital | No | No |
| 2017 | Kim | Medical costs. Cesarean delivery rates. and length of stay in specialty hospitals vs. non-specialty hospitals in South Korea | 2012 – 2014 | 34.4% - 46.7% | 418.141 | No | Database | No | Hospital category | No | No |
| 2017 | Lafitte | Rate of caesarean sections according to the Robson classification: Analysis in a French perinatal network - Interest and limitations of the French medico-administrative data (PMSI) | 2014 | 14.5% - 33.2% | 15.413 | No | Database | No | Hospital | No | No |
| 2017 | Li | Geographic Variations and Temporal Trends in Cesarean Delivery Rates in China. 2008-2014 | 2008 – 2014 | 4.0% - 62.5% | 15.123.276^[[3]](#footnote-3)^ | No | Database | No | Region | Yes | No |
| 2017 | Lundsberg | Variation in Hospital Intrapartum Practices and Association With Cesarean Rate | ? | 21.0% - 42.0% | ? | Yes | Questionnaire | No | Hospital | No | No |
| 2017 | Maeda | Cesarean section rates and local resources for perinatal care in Japan: A nationwide ecological study using the national database of health insurance claims | 2013 | 14.0% - 25.6% | 1.029.816 | No | Database | No | Region | No | No |
| 2017 | McClelland | Factors associated with cesarean delivery rates: a single-institution experience | 2005 – 2013 | 9.9% - 65.8% | 4.495 | No | Database | Yes | Individual | No | Yes |
| 2017 | Mesterton | Case mix adjusted variation in cesarean section rate in Sweden | 2011 – 2012 | 12.1% - 22.6% | 139.756 | No | Database | Yes | Hospital | Yes | No |
| 2017 | Morris | Hospital-ownership status and cesareans in the United States: The effect of for-profit hospitals | 2011 – 2012 | 28.4% - 35.8% | 856 | Yes | Questionnaire | Yes | Hospital category | No | No |
| 2017 | Mumtaz | Rising trends and inequalities in cesarean section rates in Pakistan: Evidence from Pakistan Demographic and Health Surveys. 1990-2013. | 1991 – 2013 | 29.0% - 31.2% (HC)  1.7% - 27.7% (R) | 7.446 | Yes | Questionnaire | Yes | Hospital category. Region | No | No |
| 2017 | Omani-Samani | Cesarean Section and Socioeconomic Status in Tehran. Iran. | 2015 | 62.2% - 91.7% | 4.308 | No | Questionnaire | No | Hospital category | No | No |
| 2017 | Ortiz-Prado | Cesarean section rates in Ecuador: a 13-year comparative analysis between public and private health systems | 2001 – 2013 | 22.3% - 57.5% (HC). 13.0% - 44.0% (R) | 1.796.826 | No | Database | No | Hospital category. Region | No | No |
| 2017 | Plevani | Cesarean delivery rates and obstetric culture - an Italian register-based study | 2013 | 9.9% - 86.4% (H)  25.8% - 31.0% (HC) | 87.896 | No | Database | No | Hospital. Hospital category | Yes | No |
| 2017 | Pyykonen | Cesarean section trends in the Nordic Countries - a comparative analysis with the Robson classification | 2000 – 2011 | 15.7% - 19.2% | 3.398.586 | No | Database | Yes | National | No | No |
| 2017 | Riddell | Inter-institutional Variation in Use of Caesarean Delivery for Labour Dystocia | 2008 – 2012 | 4.5% - 24.7% (H)  19.5% - 24.4% (R) | 403.205 | No | Database | Yes | Hospital. Region | No | Yes |
| 2017 | Sepehri | Regional Gradients in Institutional Cesarean Delivery Rates: Evidence from Five Countries in Asia | 2005 – 2013 | 19.1% - 54.9% | 45.119 | Yes | Questionnaire | Yes | National | No | No |
| 2017 | Shabila | Rates and trends in cesarean sections between 2008 and 2012 in Iraq | 2008 – 2012 | 9.5% - 38.9% | 1.300.103 | No | Other | No | Region | No | No |
| 2017 | Thornton | Cesarean Outcomes in US Birth Centers and Collaborating Hospitals: A Cohort Comparison | 2006 – 2011 | 4.1% - 5.0% | 11.303 | Yes | Database | Yes | Hospital category | Yes | No |
| 2017 | Vankan | Practice variation of vaginal birth after cesarean and the influence of risk factors at patient level: a retrospective cohort study | 2010 | 6.0% - 54.0% (H)  26.2% - 42.0% (HC) | 1.068 | Yes | EPF | Yes | Hospital. Hospital category | No | No |
| 2017 | Wang | Caesarean deliveries in China | 2011 | 50.9% - 55.9% | 112.138 | No | EPF | No | Hospital category | No | No |
| 2018 | Boatin | Within country inequalities in caesarean section rates: observational study of 72 low and middle income countries | 2018 | 0.6% - 58.9% (N) | ? | Yes | Questionnaire | No | National | No | No |
| 2018 | Boerma | Global epidemiology of use of and disparities in caesarean sections | 2000 – 2015 | 0.2% - 62.5% (R)  National unknown | ? | Both | Other | No | Region. National | No | Yes |
| 2018 | Bolognani | Robson 10-groups classification system to access C-section in two public hospitals of the Federal District/Brazil | 2013 | 42.3% - 50.8% | 6.579 | No | Database | No | Hospital | No | No |
| 2018 | Chi | Trends and predictors of cesarean birth in Singapore. 2005-2014: A population-based cohort study | 2005 – 2014 | 25.0% - 36.5% | 42.232 | No | Database | Yes | Hospital category | No | No |
| 2018 | Di Giovanni | Trend in primary caesarean delivery: a five-year experience in ABRUZZO. ITALY | 2009 – 2013 | 17.9% - 43.0% | 8.464 | No | Database | No | Hospital | No | No |
| 2018 | Giang | Monitoring and interventions are needed to reduce the very high Caesarean section rates in Vietnam | 2015 – 2016 | 57.9% - 70.6% | 20.535 | No | Database | No | Hospital category | No | No |
| 2018 | Maeda | Cesarean section rates and local resources for perinatal care in Japan: A nationwide ecological study using the national database of health insurance claims | 2013 | 14.0% - 25.6% | 1.029.816 | No | Database | Yes | Region | No | Yes |
| 2018 | Nakamura-Pereira | Elective repeat cesarean delivery in women eligible for trial of labor in Brazil | 2011 – 2012 | 44.1% - 95.8% | 23.894 | Yes | Database | No | Hospital category | No | Yes |
| 2018 | Norum | Caesarean Section Rates and Activity-Based Funding in Northern Norway: A Model-Based Study Using the World Health Organization's Recommendation | 2016 | 13.9% - 20.3% | 4.860 | No | Database | No | Hospital | No | No |
| 2018 | Pasko | Variation in the Nulliparous. Term. Singleton. Vertex Cesarean Delivery Rate | 2008 – 2011 | 15.0% - 35.2% | 38.275 | No | Database | Yes | Hospital | No | Yes |
| 2018 | Santas | Trends of caesarean section rates in Turkey | 1993 – 2013 | 42.4% - 66.3% (HC). 38.1% - 57.7% (R) | 2.616 | Yes | Questionnaire | No | Hospital. Hospital category | No | No |
| 2018 | Sepehri | Institutional setting and wealth gradients in cesarean delivery rates: Evidence from six developing countries | 2007 – 2015 | 19.1% - 58.6% | 51.744 | Yes | Questionnaire | Yes | National | No | Yes |
| 2018 | Singh | High prevalence of cesarean section births in private sector health facilities- analysis of district level household survey-4 (DLHS-4) of India. | 2011 | 13.7% - 37.9% | 22.111 | Yes | Questionnaire | No | Hospital category | No | No |
| 2018 | Wehberg | Risk factors and between-hospital variation of caesarean section in Denmark: a cohort study. | 2009 – 2012 | 3.6% - 10.6% | 226.612 | No | Database | Yes | Hospital | No | Yes |
| 2018 | Zimmo | Differences in rates and odds for emergency caesarean section in six Palestinian hospitals: a population-based birth cohort study. | 2015 – 2016 | 5.0% - 15.7% | 32.321 | No | Database | Yes | Hospital | No | No |
| 2018 | Zimmo | Caesarean section in Palestine using the Robson Ten Group Classification System: a population-based birth cohort study. | 2016 – 2017 | 20.6% - 24.6% | 18.908 | No | Database | No | Hospital | No | No |
| 2019 | Atuheire | Spatial and temporal trends of cesarean deliveries in Uganda: 2012-2016. | 2012 – 2016 | 8.0% - 32.0% (HC). 2.0% - 26.0% (R) | 4.038.137 | No | Database | No | Hospital category. Region | No | No |
| 2019 | Borrescio-Higa | Publicly insured caesarean sections in private hospitals: a repeated cross-sectional analysis in Chile | 2001 - 2014 | 30.9% - 77.2% | 184.787 | No | Database | Yes | Hospital category | Yes | No |
| 2019 | Duncan | Mode of delivery in pregnancies with gastroschisis according to delivery institution | 2008 – 2015 | 41.2% - 76.6% | 94 | No | Database | No | Hospital | Yes | No |
| 2019 | Eyi | An analysis of the high cesarean section rates in Turkey by Robson classification | 2017 | 39.7% - 70.6% | 887.683 | Yes | Database | No | Hospital category | No | No |
| 2019 | Giordano | The misleading choice for safer births in Brazilian's most developed region: a cross-sectional study | 2011 – 2014 | 41.6% - 90.8% | 1.276 | Yes | Other | No | Hospital category | No | No |
| 2019 | Gu | Examining Caesarean Section Rates in Canada Using the Modified Robson Classification | 2016 – 2017 | 23.2% - 35.3% | 286.201 | No | Database | No | Region | No | No |
| 2019 | Guilmoto | Trends. Regional Variations. and Socioeconomic Disparities in Cesarean Births in India. 2010-2016 | 2010 – 2016 | 5.8% - 57.7% | 259.627 | Yes | Questionnaire | No | Region | Yes | No |
| 2019 | Harrison | A Prospective. Population-Based Study of Trends in Operative Vaginal Delivery Compared to Cesarean Delivery Rates in Low- and Middle-Income Countries. 2010-2016 | 2010 – 2016 | 1.0% - 37.0% | 354.287 | No | Database | No | Region | No | No |
| 2019 | Holmer | The rate and perioperative mortality of caesarean section in Sierra Leone. | 2016 | 0.4% - 5.2% | 256.211 | No | Other | No | Region | No | No |
| 2019 | Hoxha | Health system factors and caesarean sections in Kosovo: a cross-sectional study | 2015 | 15.2% - 36.9% | 859 | Yes | Questionnaire | No | Hospital | No | No |
| 2019 | Jahnke | Rising rates of cesarean delivery in Ecuador: Socioeconomic and institutional determinants over two decades. | 1989 – 2012 | 30.0% - 64.7% | 2.725 | Yes | Questionnaire | Yes | Hospital category | No | No |
| 2019 | Kim | An ecological study of geographic variation and factors associated with cesarean section rates in South Korea | 2013 | 22.8% - 68.0% | 436.192 | No | Database | No | Region | No | Yes |
| 2019 | Korenc | Classification of Primary Caesarean Sections in Labor and its Usefulness for Analysis of Slovenian Perinatal Data | 2013 – 2014 | 4.1% - 20.9% | 13.572 | No | Database | No | Hospital | Yes | No |
| 2019 | McDonald | In States That Border Mexico. Cesarean Rates Were Highest For Hispanic Women Living In Border Counties In 2015 | 2015 | 30.9% - 38.3% | 470.484 | No | Database | Yes | Region | No | Yes |
| 2019 | Mia | Socio-demographic. health and institutional determinants of caesarean section among the poorest segment of the urban population: Evidence from selected slums in Dhaka. Bangladesh | 2014 – 2016 | 24.0% - 73.0% (HC). 6.9% - 35.3% (R) | 1.063 | Yes | Questionnaire | Yes | Hospital category. Region | No | No |
| 2019 | Ming | Dissecting the current caesarean section rate in Shanghai. China. | 2016 – 2016 | ?^[[4]](#footnote-4)^ | 10.751 | Yes | EPF | No | Hospital category | No | No |
| 2019 | Panda | Rising extent of caesarean delivery and its differential access in regions of India 2005–2016. | 2000 – 2016 | 9.2% - 36.8% | ? | Yes | Questionnaire | No | Region | No | No |
| 2019 | Srivastava | Exploring the spatial patterns of cesarean section delivery in India: Evidence from National Family Health Survey-4. | 2005 – 2016 | 11.9% - 40.9% (HC). 5.8% - 58.0% (R) | 255.726 | Yes | Questionnaire | Yes | Hospital category. region | No | Yes |
| 2019 | Uribe-Leitz | Trends of caesarean delivery from 2008 to 2017. Mexico | 2008 – 2017 | 28.0% - 79.8% (HC). 31.1% - 52.1% (R) | 2.064.507 | No | Database | No | Hospital category. region | No | No |
| 2019 | Wondie | Cesarean delivery among women who gave birth in Dessie town hospitals. Northeast Ethiopia | 2013 | 18.2% - 76.1% | 512 | Yes | Other | No | Hospital category | No | No |
| 2019 | Yisma | Cesarean section in Ethiopia: prevalence and sociodemographic characteristics. | 2000 – 2016 | 6.5% - 23.0% (HC). 0.4% - 21.4% (R) | 11.023 | Yes | Questionnaire | Yes | Hospital category. region | No | No |
| 2019 | Zahumensky | Evaluation of cesarean delivery rates at three university hospital labor units using the Robson classification system | 2017 | 14.6% - 42.8% | 8.237 | No | EPF | No | Hospital | Yes | No |
| 2019 | Zaiden | Influence of hospital characteristics on the performance of elective cesareans in Southeast Brazil | 2011 – 2012 | 28.3% - 83.2% | 10.155 | Yes | Other | Yes | Hospital category | No | Yes |
| 2020 | Cegolon | Understanding Factors Leading to Primary Cesarean Section and Vaginal Birth After Cesarean Delivery in the Friuli-Venezia Giulia Region (North-Eastern Italy). 2005-2015 | 2005 – 2015 | 15.2% - 34.3% | 109.246 | No | Database | Yes | Hospital | No | No |
| 2020 | Iobst | Variation of intrapartum care and cesarean rates among practitioners attending births of low-risk. nulliparous women | 2002 – 2007 | 0.0% - 53.3% (I). 7.2% - 18.9% (H) | 13.196 | No | Database | Yes | Individual. hospital | No | No |
| 2020 | Karalasingam | Caesarean section rates from Malaysian tertiary hospitals using Robson's 10-group classification | 2011 – 2015 | 17.0% - 32.0% | 130.738 | No | Database | No | Hospital | No | No |
| 2020 | Kumar | Household- and community-level determinants of low-risk Caesarean deliveries among women in India | 2010 – 2016 | 13.2% - 43.1% (HC). 14.5% - 39.9% (R) | 59.318 | Yes | Questionnaire | No | Hospital category. region | No | No |
| 2020 | Seidu | Not just numbers: beyond counting caesarean deliveries to understanding their determinants in Ghana using a population based cross-sectional study | 2009 – 2014 | 15.4% - 18.5% (HC). 8.1% - 25.1% (R) | 2.742 | Yes | Questionnaire | No | Hospital category. region | No | No |
| 2020 | Vanderlaan | Geospatial variation in caesarean delivery. | 2008 – 2012 | 12.0% - 31.7% | ? | No | Database | No | Region | No | No |

1. Approximation based on Figure 2 [↑](#footnote-ref-1)
2. Public/private hospital caesarean section rate is not stratified without public/private funding [↑](#footnote-ref-2)
3. Largest study in database: cohort size 100.873.051 (2008 – 2014). [↑](#footnote-ref-3)
4. Only stratified by Robson category [↑](#footnote-ref-4)
