# Supplementary material for: Design characteristics of studies on medical practice variation of caesarean section rates: a scoping review
Source: BMC Pregnancy Childbirth. 2020 Aug 20;20:478. doi: 10.1186/s12884-020-03169-3 (PMC7441547; doi:10.1186/s12884-020-03169-3)
Supplement: Supplementary file 3 — Additional file 3. Studies per country. Additional file 3 describes the number of studies on medical practice variation of caesarean section rates that were conducted in each country. [file 12884_2020_3169_MOESM3_ESM.docx]

# Additional file 3: Studies per country

| Afghanistan | 1 |
| --- | --- |
| Angola | 1 |
| Arab world | 1 |
| Argentina | 5 |
| Australia | 17 |
| Austria | 3 |
| Bangladesh | 6 |
| Belgium | 6 |
| Benin | 1 |
| Brazil | 22 |
| Burkina Faso | 3 |
| Burundi | 2 |
| Cambodia | 3 |
| Cameroon | 2 |
| Canada | 15 |
| Chad | 1 |
| Chile | 3 |
| China | 13 |
| Congo | 4 |
| Cuba | 2 |
| Cyprus | 1 |
| Czech Republic | 3 |
| Denmark | 9 |
| Dominican Republic | 1 |
| Ecuador | 6 |
| Egypt | 4 |
| Estonia | 1 |
| Ethiopia | 5 |
| Finland | 5 |
| France | 6 |
| Georgia | 2 |
| Germany | 8 |
| Ghana | 3 |
| Greece | 3 |
| Guatemala | 2 |
| Guinea | 1 |
| Honduras | 1 |
| Hong Kong | 1 |
| Hongary | 3 |
| Iceland | 3 |
| India | 15 |
| Indonesia | 2 |
| Iran | 5 |
| Iraq | 1 |
| Ireland | 8 |
| Israel | 1 |
| Italy | 14 |
| Ivory Coast | 1 |
| Japan | 7 |
| Jordan | 3 |
| Kenya | 7 |
| Korea | 1 |
| Kosovo | 1 |
| Latin America | 1 |
| Latvia | 1 |
| Lesotho | 2 |
| Libanon | 4 |
| Lithuania | 1 |
| Luxembourg | 1 |
| Madagascar | 2 |
| Malawi | 2 |
| Malaysia | 3 |
| Mali | 2 |
| Malta | 1 |
| Mexico | 8 |
| Mongolia | 0 |
| Mozambique | 3 |
| Nepal | 5 |
| New Zealand | 3 |
| Nicaragua | 4 |
| Niger | 3 |
| Nigeria | 4 |
| Norway | 8 |
| Pakistan | 7 |
| Palestina | 3 |
| Paraguay | 4 |
| Peru | 5 |
| Philippines | 6 |
| Poland | 2 |
| Portugal | 5 |
| Puerto Rico | 1 |
| Qatar | 1 |
| Romania | 1 |
| Rwanda | 3 |
| Saudi Arabia | 1 |
| Scotland | 2 |
| Senegal | 3 |
| Sierra Leone | 3 |
| Singapore | 1 |
| Slovakia | 4 |
| Slovenia | 4 |
| South Africa | 1 |
| South Korea | 2 |
| Spain | 7 |
| Sri Lanka | 3 |
| Sweden | 8 |
| Switserland | 2 |
| Taiwan | 4 |
| Tanzania | 2 |
| Thailand | 5 |
| the Netherlands | 6 |
| Turkey | 4 |
| Uganda | 5 |
| United Kingdom | 13 |
| United States | 75 |
| Uruguay | 1 |
| Venezuela | 1 |
| Vietnam | 4 |
| World | 9 |
| Zambia | 2 |
| Zimbabwe | 4 |
