# Supplementary material for: Design characteristics of studies on medical practice variation of caesarean section rates: a scoping review
Source: BMC Pregnancy Childbirth. 2020 Aug 20;20:478. doi: 10.1186/s12884-020-03169-3 (PMC7441547; doi:10.1186/s12884-020-03169-3)
Supplement: Supplementary file 4 — Additional file 4. Variables. Additional file 4 summarizes all the variables that were used in the included studies: - Patient characteristics: variables that were used as baseline characteristic or for case-mix correction. - Outcome: variables on maternal and neonatal outcome. - Organization or physician characteristics: characteristics that were used as determinant to explain differences in caesarean section rates. [file 12884_2020_3169_MOESM4_ESM.docx]

# Additional file 4: Variables

**Patient characteristics (maternal)**

| Abnormal cervix/vagina | 1 |
| --- | --- |
| Abnormal uterine neck | 1 |
| Age | 118 |
| Age at marriage | 2 |
| Age of husband | 2 |
| Anaemia | 7 |
| APLA | 1 |
| Astma/COPD | 1 |
| Autoimmune disease | 1 |
| BMI | 23 |
| Cardiovascular disease | 7 |
| Cardiovascular renal disease | 2 |
| Caste / tribes | 1 |
| Cerebral occlusion / haemorrhage | 2 |
| Charlson index | 1 |
| City of origin | 1 |
| Classical caesarean scar | 1 |
| Coagulation disorder | 1 |
| Cohabitation / marital status | 25 |
| Comorbidity | 11 |
| Country of origin | 19 |
| Cyst | 1 |
| Deprivation index | 1 |
| Diabetes (1/2/gestational) | 44 |
| Duration membranes broken | 1 |
| Duration taking balanced diet | 1 |
| Drug usage (ie cocaine) | 1 |
| Dyskinesia | 1 |
| Education (father) | 2 |
| Education (mother) | 48 |
| Employment status (father) | 3 |
| Employment status (mother) | 9 |
| Epilepsy | 2 |
| Fever | 2 |
| Haemoglobinopathies | 1 |
| Height | 7 |
| Hemoglobin | 1 |
| Hepatosis | 2 |
| Hypertension | 41 |
| Inflamatory Bowel Disease | 1 |
| Income / wealth status | 23 |
| Infertility | 1 |
| Insurance status | 19 |
| Iso-umminisation rH antigen | 2 |
| Language | 1 |
| Locality | 14 |
| Low-risk pregnancy | 1 |
| Low urinary tract infecton | 3 |
| Lupus or sclerodermia | 1 |
| Malaria | 1 |
| Malignancy | 2 |
| Maternal heart rate | 1 |
| Maternal infection | 2 |
| Myomectomy | 1 |
| NGO membership | 1 |
| No complication | 1 |
| Occupation | 3 |
| Other current conditions classified elsewere | 1 |
| Other or unspecified abnormality of organs or soft tissue of pelvis | 1 |
| Other specified infectious and parasitic diseases | 1 |
| Other venous complication | 1 |
| Parity | 74 |
| Participation in NCMS (incentive scheme) | 2 |
| Payment method | 7 |
| Pelvic condition | 4 |
| Perinatal death (history of) | 3 |
| Place of residence (urban/rural) | 8 |
| Psychiatric disease | 1 |
| Pulmonal pathology | 6 |
| Pyelonefritis | 1 |
| Race / skin color | 29 |
| Religion | 1 |
| Renal disease | 6 |
| Sexual transmitted disease | 11 |
| Sicle cell anaemia | 1 |
| Smoking | 15 |
| Substance abuse | 3 |
| Thyroid disease | 5 |
| Tromboflebitis | 1 |
| Tuberculosis | 1 |
| Tumors of body uterus | 1 |
| Use of assisted reproductive technology | 8 |
| Vaginal discharge | 1 |
| Vasculo-renal syndrome | 2 |
| Weight | 8 |
| Weight gain | 5 |

**Patient characteristics (obstetrics)**

| (Women reported reason:) labour complications | 4 |
| --- | --- |
| Abnormal rest after birth | 1 |
| Abortion (threats) | 4 |
| Adverse outcome in previous pregnancy | 2 |
| Analgesia, type of | 2 |
| Artificial rupture membranes | 1 |
| Augmentation of labour | 6 |
| Birth order | 1 |
| Birthweight | 61 |
| Caesarean section without indication | 1 |
| Cephalopelvic disproportion | 2 |
| Cervical dilatation at admission | 3 |
| Cervical effacement at admission | 1 |
| Cervical insufficiency | 3 |
| Chorioamnionitis | 10 |
| Complete fetal heart block | 1 |
| Complications in (previous) pregnancy / labour (composite) | 12 |
| Congenital anomaly | 9 |
| Cord entanglement | 2 |
| Cord prolaps | 9 |
| Day of birth | 4 |
| Duration of labour | 5 |
| Duration of labour, stage 1 | 2 |
| Duration of labour, stage 2 (for instrumental delivery) | 1 |
| Duration of labour, stage 2 | 2 |
| Duration of Oxytocin | 1 |
| Duration or labour, stage 2 (for cs) | 1 |
| Dystocia | 13 |
| Eclampsia | 14 |
| Failed induction | 1 |
| Fetal distress | 19 |
| Fetal growth restriction | 17 |
| Fetal macrocephaly | 1 |
| Fetal monitoring | 1 |
| Fetal station at admission | 1 |
| Fetal supraventricular tachycardy | 1 |
| First degree laceration | 1 |
| Funic presentation | 1 |
| Gestational age (including preterm delivery) | 66 |
| Gravidity | 1 |
| Haemorrhage | 1 |
| Head circumference | 3 |
| High risk pregnancy | 2 |
| High vaginal laceration | 1 |
| Hospital / clinical birth | 4 |
| Hospital admission | 4 |
| In utero transfer | 1 |
| Induction of labour | 6 |
| Instrumental delivery (history of / applied) | 7 |
| Intensive care admission | 1 |
| Inter pregnancy interval | 1 |
| Iso-umminisation rH antigen | 2 |
| Lamaze training | 1 |
| Last antenatal check-up showed abnormality | 1 |
| Length of baby | 1 |
| Length of hospital stay | 1 |
| Maximum dose of Oxytocin | 1 |
| Meconium | 3 |
| Miscarriage (history of) | 4 |
| (Monoamniotic) twins | 2 |
| Multiple pregnancy | 41 |
| Neonatal status | 1 |
| Non-CS scar | 2 |
| Number of ultrasonic examinations | 3 |
| Obstetric risk | 1 |
| Obstructed labour | 1 |
| Oligohydramnion | 16 |
| Onset of labour | 17 |
| Other problems of amnios | 2 |
| Other specified indication for care or intervention related to labor and delivery | 1 |
| Oxytocine usage beginning cervical dilatation (cm) | 1 |
| Partogram monitor | 1 |
| Patient preference of mode of birth | 1 |
| Pelvic floor/perineal/birth canal injuries | 1 |
| Placenta pathology | 8 |
| Placenta praevia | 20 |
| Placental abruption | 17 |
| Placental weight | 1 |
| Planned pregnancy | 1 |
| Polyhydramnion | 13 |
| PPROM | 13 |
| Pre-eclampsia/HELLP | 26 |
| Precipitous labour | 1 |
| (Pre-delivery) lenght of stay (LoS) | 2 |
| Pregnancies not suitable for trial of labour | 1 |
| Pregnancy induced senseless | 1 |
| Prenatal visits | 13 |
| Prepartal stay in days | 1 |
| Presentation | 45 |
| Previous preterm birth | 1 |
| Primary section | 1 |
| Prior 3th or 4th degree tear | 1 |
| Prior non progressive labor | 1 |
| Prior pregnancy loss | 1 |
| Private obstetric care | 2 |
| (no of) Prior vaginal delivery | 3 |
| Reduced fetal movement | 1 |
| Referral status | 1 |
| Regional anesthesia | 8 |
| Repeat caesarean section | 41 |
| Repeat SC, number of | 2 |
| Risk stratification (pregnancy) | 1 |
| Rupture of membranes before labour | 2 |
| Second degree laceration | 1 |
| Sex of baby | 7 |
| Stillbirth (history of) | 6 |
| Supervision of high risk pregnancy | 1 |
| Symphysiotomy (history of) | 1 |
| Third degree laceration | 1 |
| Time of birth | 3 |
| Time from admission to delivery | 1 |
| Trimester prenatal care began | 8 |
| Type of anethesia | 2 |
| Type of healthcare attendent | 2 |
| Type of pregnancy | 1 |
| Type of prenatal care received | 12 |
| Unskilled / nonprofessional | 1 |
| Use of Oxytocin/Prostaglandin | 4 |
| Uterine rupture | 1 |
| Vaginal bleeding | 21 |
| Volume of deliveries (during shift) | 1 |
| Women reported reason: less pain | 1 |
| Women reported reason: pregnancy complications | 2 |
| Women reported reason: safer | 1 |
| Women reported reason: select day of delivery | 1 |
| Women reported reason: tubal ligations | 1 |
| Working in third trimester | 1 |
| Year of delivery | 2 |

**Outcome (maternal)**

| Abortive outcome | 1 |
| --- | --- |
| Blood transfusion | 2 |
| Chorioamnionitis | 1 |
| Composite outcome | 5 |
| Endometritis | 1 |
| Episiotomy | 1 |
| Fever | 1 |
| Haemorrhage | 6 |
| Infection | 4 |
| Laceration | 2 |
| Lenght of stay | 3 |
| Mortality | 13 |
| Postpartum transfusion | 1 |

**Outcome (neonatal)**

| Apgar score | 25 |
| --- | --- |
| Apnea | 1 |
| Asphyxia | 3 |
| Birth weight | 3 |
| Bowel ischemia | 1 |
| Breastfeeding at discharge | 1 |
| Bronchopulmonary dysplasia | 1 |
| Composite outcome | 3 |
| Days (no) in obstetric ward | 1 |
| Encephalopathy | 1 |
| Erb's palsy | 1 |
| Grunting / persistent tachypnea | 2 |
| Hyperbilirubinemia | 1 |
| Hypocalcemia | 1 |
| Hypoglycemia | 1 |
| Intravenricular haemorrhage | 2 |
| Lenght of stay (NICU) | 3 |
| Lenght of ventilation use | 1 |
| Meconium aspiration syndrome | 3 |
| Mortality | 32 |
| NICU admission | 12 |
| Pneumonia | 1 |
| Pneumothorax | 1 |
| Respiratory distress syndrome | 2 |
| Resuscitation | 2 |
| Seizures | 2 |
| Sepsis | 2 |
| Sex of baby | 1 |
| Shoulder dystocia | 1 |
| Stillborn | 3 |
| Umbilical cord pH | 4 |
| Ventilatory assistance | 3 |
| Wet lung | 1 |

**Organisation characteristics (determinant)**

| % for profit beds | 1 |
| --- | --- |
| 24-h in house service | 1 |
| Adult IC available | 1 |
| Anethesia service (24h available) | 3 |
| Annual revenue / staff ratio | 1 |
| Antibiotics, dose | 1 |
| Antibiotics, given | 1 |
| Antibiotics, indication | 1 |
| Antibiotics, when | 1 |
| Antibiotics, which | 1 |
| Average GDP | 5 |
| Average hospital charge | 3 |
| Average income per capita | 2 |
| Baby friendly hospital | 1 |
| Basic emergency obstetric care facilities | 1 |
| Bed turnover rate | 1 |
| Birth in large hospital | 1 |
| Birth volume | 12 |
| Birth volume | 4 |
| Birth volume per staff | 1 |
| Complication rate | 1 |
| Complexity of hospital | 6 |
| Comprehensive emergency obstetric care facilities | 1 |
| Cost of delivery | 1 |
| Day of admission | 1 |
| Day of delivery | 3 |
| Delivery beds per 100.00 capita | 1 |
| Education | 1 |
| Epidural rate | 2 |
| Ethnicity per hospital | 1 |
| External cephanic version available | 1 |
| Female literacy | 1 |
| Fetal monitoring (available) | 1 |
| GDP spent on health | 1 |
| Gender of admission | 1 |
| Geographic location | 7 |
| Gynaecologists per 100.000 capita | 4 |
| Hospital (medicaid) births | 2 |
| Hospitalists / 1000 births | 1 |
| Hospital overall revenu | 1 |
| Hospital ownership | 8 |
| Hospital size | 8 |
| In-house paediatrics | 1 |
| In-house gynaecologist (FMF specialty) | 1 |
| Incentive | 1 |
| Indication for induction | 1 |
| Inpatient number/bed ratio | 1 |
| Instrumental birth rate | 2 |
| Insurance coverage | 1 |
| Intra-uterine pressure catheter | 1 |
| Lenght of stay in hospital | 2 |
| Level of perinatal care (availableper medical area) | 2 |
| Level of nursery | 1 |
| Low risk birth available/rate | 2 |
| Malpractice | 1 |
| Maximum motevideo units achieved | 1 |
| Maximum Oxytocine dose used | 1 |
| Mean revenue per caesarean section | 1 |
| Median nights hospitalized postpartum | 1 |
| Medicaid inpatient days | 1 |
| Medical staff/bed ratio | 1 |
| Medical staff/delivery ratio | 2 |
| Midwives per 100.000 capita | 3 |
| (National) health system available | 1 |
| Newborn care units with intubators | 1 |
| Newborn resurcitation available | 1 |
| (no of) NICU beds (per birth) available | 2 |
| Night delivery | 1 |
| Number of family practice residents | 1 |
| Number of fulltime faculty staff | 1 |
| Number of general medical doctors | 2 |
| Number of hospitals | 4 |
| Number of hospitals able to provide CS | 2 |
| Number of midwives | 2 |
| Number of nulliparous deliveries | 1 |
| Number of (obstetric) beds | 3 |
| Number of obstetric staff | 8 |
| Number of operative deliveries | 1 |
| Number of residents | 2 |
| Number of staff (general) | 1 |
| Number of women in reproductive age | 2 |
| Number of physicians per 1.000 deliveries / physician density | 3 |
| Nurse-to-patient-ratio | 1 |
| Obstetric transfusions | 1 |
| Obstetric training provision | 1 |
| Overall inpatients per month | 1 |
| Overall outpatient per day | 1 |
| Oxytocine (augmentation) | 2 |
| Percentage birth attended by certified midwives/doctor | 2 |
| Percentage birth with regional anesthesia | 2 |
| Percentage government deliveries | 1 |
| Percentage induction/augmentation of labour | 5 |
| Percentage insitutional deliveries | 3 |
| Percentage of caesarean sections with general anesthesia | 2 |
| Percentage private patients | 1 |
| Percentage of urban population | 2 |
| Percentage of VBAC breech | 1 |
| Physician age | 1 |
| Physicians' gender | 1 |
| Policy to regulate caesarean sections | 1 |
| Population density | 1 |
| Population served per hospital | 2 |
| Prenatal care coverage | 1 |
| Prenatal electronic medical record | 1 |
| Primary care doctors per 100.000 capita | 2 |
| Private/public hospital | 6 |
| Profit status of hospital | 1 |
| Proportion of women with mechanical dystocia | 1 |
| Residency program available | 10 |
| Shift delivered | 1 |
| Short-term hospitald | 1 |
| (Skilled) birth attendance | 3 |
| Social security | 1 |
| Staff / bed ratio | 1 |
| (No of) staff delivering > 10 deliveries | 1 |
| Structured patient review, existence | 1 |
| Subspecialty of doctor attending delivery | 1 |
| Teaching status hospital | 1 |
| Time of delivery | 2 |
| Total fertility rate | 3 |
| Total population | 6 |
| Type of hospital | 2 |
| Usage of fetal scalp electrode | 1 |
| VBAC allowed | 1 |
| Weekend delivery | 2 |
| Year of delivery | 1 |

**Physician characteristics (determinant)**

| Age | 3 |
| --- | --- |
| Annual malpractice premium | 1 |
| Board cerfification | 1 |
| Gender | 2 |
| Graduation from Liaison Committee | 1 |
| Operative vaginal delivery rate | 1 |
| Proportion of high risk deliveries | 1 |
| Solo/group practice | 2 |
| Specialty | 2 |
| Total number of deliveries | 2 |
| University residency | 1 |
| Years of experience | 5 |
